# The impact of vitamin D pathway genetic variation and circulating 25-hydroxyvitamin D on cancer outcome: systematic review and meta-analysis

**DOI:** 10.1038/bjc.2017.44

**Published:** 2017-03-16

**Authors:** P G Vaughan-Shaw, F O'Sullivan, S M Farrington, E Theodoratou, H Campbell, M G Dunlop, L Zgaga

**Affiliations:** 1 MRC Human Genetics Unit, Institute of Genetics and Molecular Medicine, University of Edinburgh, Edinburgh EH42XU, UK; 2 Department of Public Health and Primary Care, Trinity College Dublin, Dublin 24, Republic of Ireland; 3 Centre for Population Health Sciences, University of Edinburgh, Edinburgh EH164UX, UK

**Keywords:** cancer, survival, vitamin D receptor, SNP, 25-hydroxyvitamin D

## Abstract

**Background:**

Vitamin D has been linked with improved cancer outcome. This systematic review and meta-analysis investigates the relationship between cancer outcomes and both vitamin D-related genetic variation and circulating 25-hydroxyvitamin D (25OHD) concentration.

**Methods:**

A systematic review and meta-analysis of papers until November 2016 on PubMed, EMBASE and Web of Science pertaining to association between circulating vitamin D level, functionally relevant vitamin D receptor genetic variants and variants within vitamin D pathway genes and cancer survival or disease progression was performed.

**Results:**

A total of 44 165 cases from 64 studies were included in meta-analyses. Higher 25OHD was associated with better overall survival (hazard ratio (HR=0.74, 95% CI: 0.66–0.82) and progression-free survival (HR=0.84, 95% CI: 0.77–0.91). The rs1544410 (BsmI) variant was associated with overall survival (HR=1.40, 95% CI: 1.05–1.75) and rs7975232 (ApaI) with progression-free survival (HR=1.29, 95% CI: 1.02–1.56). The rs2228570 (FokI) variant was associated with overall survival in lung cancer patients (HR=1.29, 95% CI: 1.0–1.57), with a suggestive association across all cancers (HR=1.26, 95% CI: 0.96–1.56).

**Conclusions:**

Higher 25OHD concentration is associated with better cancer outcome, and the observed association of functional variants in vitamin D pathway genes with outcome supports a causal link. This analysis provides powerful background rationale to instigate clinical trials to investigate the potential beneficial effect of vitamin D in the context of stratification by genotype.

The importance of vitamin D for bone health is well established, but the role of vitamin D beyond the skeletal system has been under debate for decades (Theodoratou *et al*, 2014). In recent years, it has become apparent that the vitamin D receptor (VDR) is expressed in most cells, and that multiple tissues have the ability to convert the primary circulating form of vitamin D into the active form (Bouillon *et al*, 2013), implying that extra-skeletal effects of vitamin D are likely.

While typically thought of as ‘vitamin', it may be more appropriate to regard the primary circulating form, 25-hydroxyvitamin D (25OHD), as a pre-hormone and the primary active form, 1,25-dihydroxyvitamin D (1,25(OH)_2_D), as a *hormone*. It has been previously recognised that mutations in genes involved in response to hormones, their metabolism or actions may affect the prognosis of disease and thus act as modifiers. Correspondingly, 1,25(OH)_2_D binds to the VDR (a ligand-dependent transcription factor) and polymorphisms in the *VDR* gene have been shown to modify the activity of this VitD–VDR complex (Anderson *et al*, 2003): for example, rs11568820 is situated in the *VDR* promoter region and can influence transcriptional activity (Yamamoto *et al*, 1999), while rs2228570 affects the translational start site (Uitterlinden *et al*, 2004). Therefore, it is hypothesised that not only vitamin D status but also expression and structure of *VDR* determine molecular actions, and can potentially modify cancer risk and survival (Flugge *et al*, 2007; Li *et al*, 2007; Zgaga *et al*, 2014). The VitD–VDR complex has the ability to exert downstream biological effects; amongst others, it can regulate the expression of multiple target genes, including several with anti-tumour properties (Ramagopalan *et al*, 2010). Moreover, polymorphisms in the *VDR* gene have been linked to cancer risk, including prostate (Taylor *et al*, 1996), breast (Lowe *et al*, 2005), skin and bowel (Ingles *et al*, 2001; Xu *et al*, 2014; Serrano *et al*, 2016), and VDR expression has been linked to survival in prostate and breast cancer (Berger *et al*, 1991; Hendrickson *et al*, 2011; Ditsch *et al*, 2012). Unlike highly variable environmental exposures (sunlight, diet and supplements) or seasonally varying 25OHD levels (Kelly *et al*, 2015), genetic variants are constant, present since conception and cannot be modified by the disease; thereby removing reverse causation concerns.

Three aspects further strengthen the case for understanding the associations between vitamin D and cancer outcomes: first, cancer incidence and mortality are increasing (CRUK, 2015); second, vitamin D deficiency is common worldwide among otherwise healthy individuals (Holick, 2007; Zgaga *et al*, 2011), and particularly among cancer patients (Crew *et al*, 2009; Fakih *et al*, 2009; Shanafelt *et al*, 2011; Vrieling *et al*, 2011; Zgaga *et al*, 2014), and third, vitamin D deficiency is a modifiable risk factor; based on the studies that report an association between vitamin D deficiency and poorer cancers outcomes. Unsurprisingly, it has been proposed that vitamin D may have potential value as an adjuvant chemotherapeutic agent, particularly since vitamin D supplements are cheap, safe and readily available (Newton-Bishop *et al*, 2009, 2015; Drake *et al*, 2010; Hatse *et al*, 2012; Zgaga *et al*, 2014).

Here we present a systematic review and meta-analysis examining the role of vitamin D on cancer progression and survival. We conducted a comprehensive evaluation of the literature that examines the associations between cancer outcomes and genetic factors involved in the vitamin D pathway, in addition to circulating 25OHD concentration. Focus on vitamin D-related genetic variation allowed us to partially mitigate against potential confounding or reverse causation, biases that typically limit implications of findings from observational vitamin D studies.

## Materials and methods

### Literature search

We performed a systematic literature review and meta-analysis following PRISMA guidelines (Moher *et al*, 2009). The electronic databases PubMed (NCBI, 2015), EMBASE (EMBASE, 2015), and Web of Science (JISC, 2015) were searched up to week 3, November 2015. We searched for studies that examined the association between cancer outcomes and (i) measured vitamin D levels and (ii) genetic factors known to affect vitamin D metabolism or pathways. A list of search terms was compiled using a number of core papers in the field. For cancer outcomes, we included a combination of terms: cancer, neoplasm, malignant, malignancy with survival, outcome, prognosis, mortality, death, recurrence. For vitamin D levels, we included terms: 25-hydroxyvitamin D, calcidiol and 25OHD; for vitamin D receptor, and for commonly studied variants, we searched for: vitamin D receptor, *VDR*, rs1544410, *BsmI*, rs10735810, rs2228570, *FokI*, rs7975232, *ApaI*, rs11568820, *Cdx-2*, rs2282679, rs12785878, rs10741657 and rs6013897. Finally, we also included variation in genes related to vitamin D synthesis, transport or metabolism: 1-*α*-hydroxylase, *CYP27B1*, 25-hydroxylase, *CYP2R1*, 24-hydroxylase, *CYP24A1*, vitamin D binding protein, 27-hydroxlyase and *CYP27A1*. Genetic variants beyond those explicitly searched for were only included if previously shown to affect vitamin D metabolism. We considered all human research full text articles, with no restriction on language or article type. Bibliographies of retrieved papers and previous reviews were hand-searched to identify other relevant studies.

### Selection criteria and selection of relevant studies

Study inclusion ‘PICO' criteria were as follows: (i) participants: individuals of any age who received a diagnosis of cancer; (ii) intervention/Exposures: assessment of vitamin D status or genetic factors known to affect vitamin D concentration, metabolism or pathways; (iii) comparators: study reports a quantitative association between cancer outcome and either vitamin D status (e.g., concentration, quartiles, low/high levels) sampled at most 1 year prior to the diagnosis, or any germline genetic variation or gene expression in normal tissue; and (iv) Outcome: cancer-specific or all-cause mortality, or disease progression (e.g., disease-free survival, local recurrence or metastasis). Observational retrospective and prospective cohorts were included.

In relation to patients, exclusion criteria were: (i) pre-cancerous lesions, and (ii) mixed-cancer cohort without site-specific reporting; in relation to exposures: (iii) vitamin D intake and supplementation, (iv) acquired non-germline mutations or tumour gene expression, and (v) predicted vitamin D status; in relation to outcomes: (vi) prognostic markers such as Prostate Specific Antigen or Breslow thickness, (vii) population cancer mortality rates; in relation to study/publication type: (viii) ecological studies, and (ix) reviews, editorials, case reports, conference abstracts and nonclinical publications. If the same patient cohort was reported on more than once, we used the highest quality, largest sample size or most recent publication. Article titles and abstracts were screened for eligibility, independently by two authors (PVS and LZ or FOS). Disagreements were resolved by discussion and review of full text.

### Data extraction

The data extraction was performed by a single investigator (PVS or FOS) using the predefined data fields and extraction was cross-checked by a second investigator in its entirety (FOS or PVS). The data from eligible studies were extracted using a tailored data extraction form that included the following information: first author, publication year, location or ethnicity of patients, sample size, mean age, gender, cancer site (subtype/histology where relevant), cancer stage, any interventions (e.g., chemotherapy), vitamin D exposure studied and important meta-data (time of sampling, mean/median 25OHD values or range for categories being compared; SNP position, name and rs ID, genotypes compared and model: additive, recessive or dominant), covariates considered, details of outcomes studied, and follow-up time. Finally, hazard ratios (HR) and 95% confidence intervals (95% CIs) adjusted for the maximum number of confounding variables were extracted. We preferentially focused on cancer-specific mortality, but if these data were not available, all-cause mortality was used instead. Relative risk estimates (RR) or adjusted odds ratios (OR) were extracted where HR were not given and used in meta-analysis (Symons and Moore, 2002). Study authors were contacted to provide additional information where needed.

### Quality assessment

The methodological quality of all studies included in the systematic review was performed using the Newcastle-Ottawa Quality Assessment Scale (NOS; Wells *et al*, 2000). Two investigators (PVS and FOS) applied predefined NOS criteria to each study to generate summary quality judgement. The risk of bias was considered ‘low' for studies with score of 7 or 8; ‘unclear' for score of 5 or 6, and ‘high' for score of 4 or lower.

### Exposure assessment

The association between circulating 25OHD and outcomes was summarised in meta-analyses by comparing the risk in the highest to the lowest reported category. The majority of studies used vitamin D categories such as quartiles or tertiles. To enable inclusion of studies that used 25OHD as a continuous variable, we sought to transform the ‘continuous HR' into a ‘HR per 10 ng ml^−1^' (Box 1).

#### Genetic factors

For SNPs, the rs number naming convention was typically used in the paper and some recoding was needed to ensure that uniform reference system was followed. For example, where a restriction fragment length polymorphism was referenced, the mutation and risk allele were recoded (e.g., *FokI f* allele was converted to the rs2228570 *T* allele). The genome browser ENSEMBL (80 GRCh38.p2) was used to determine if alias names existed (e.g., *FokI,* rs10735810 and rs2228570 are the same variant). HR values were inverted where needed, so that the same allele acted as the reference. Where additive models were used, the HR values were squared in order to approximate the HR value for comparison between two homozygotes.

### Statistical analysis

We conducted meta-analyses for a range of exposure-outcome pairs by cancer site and across all sites. A meta-analysis was performed if at least two studies considered the same exposure-outcome pair. The same study may have been included multiple times in different meta-analyses if it reported on multiple subpopulations, outcomes, and/or exposures. The extracted HRs and 95% CIs were used to calculate the pooled HR estimates. The standard errors (s.e.) were used to calculate weighting for each study. The DerSimonian and Laird random-effects model was used to calculate pooled HR because of the *a priori* expected heterogeneity between studies, due to differences among populations and methodological dissimilarities between studies; most notably, different definition of 25OHD categories. All analyses were performed in R (R Core Team, 2013), and the R-package ‘metafor' was used for meta-analyses (Viechtbauer and Cheung, 2010). *P*-value <0.05 was considered statistically significant.

In order to assess the impact of study quality on results, meta-analyses were rerun (i) after exclusion of studies at high risk of bias, (ii) limited to studies at low risk of bias only, (iii) limited to studies that looked at cancer-specific mortality, (iv) excluding studies that used 25OHD as a continuous variable, and (v) excluding studies that reported RR or OR. The *I*^2^ statistic was calculated to quantify the degree of heterogeneity between studies and assess impact on the meta-analysis (Higgins *et al*, 2003). To further explore this issues arising due to the striking differences in 25OHD category definition, we conducted a stratified analysis (Cochrane, 2011) according to: (vi) the difference in mean/median 25OHD between ‘high' and ‘low' categories compared (below or ⩾20 ng ml^−1^), and (vii) the degree of deficiency in ‘low' category (mean/median 25OHD concentration below or ⩾12.5 ng ml^−1^). Publication and selection bias was investigated by checking for asymmetry in the funnel plots and running the Egger's regression test (Sterne and Egger, 2001).

## Results

**Figure 1:**
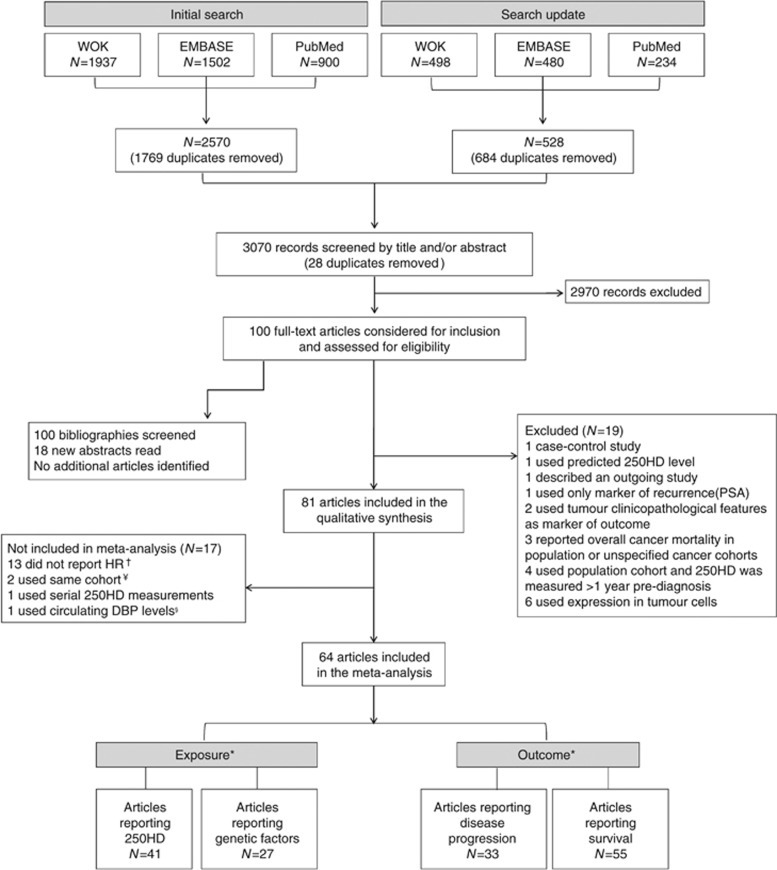
**PRISMA Flowchart of the study selection process.**Two studies used the same prostate cancer cohort but one reported on circulating 25OHD and the other on genetic variants, and so both were retained. (Holt *et al*,2010, 2013) Three publications used the same initial cohort of lung cancer patients but two reported on different subpopulations of patients (according to disease stage) and so were retained, (Zhou *et al*, 2007; Heist *et al*, 2008), while a third reported on different exposures to the first two and so was also retained (Zhou *et al*, 2006). Finally, four studies reported on the same melanoma patient cohort (Newton-Bishop *et al*, 2009, 2015; Field *et al*, 2013; Davies *et al*, 2014) (one paper scored lower in NOS scoring was excluded (Field *et al*, 2013), while the remaining three, which reported different exposure or outcomes were retained. ^§^Only a single study reported impact of circulating vitamin D-binding protein levels on outcome and so could not be included in the meta-analysis. *Includes only exposures and outcomes included in MA. Articles may report on multiple exposure-outcome pairs hence the sum of the pairs is greater than the number of articles included. For example, several papers studied the effect of more than one SNP for example, Zgaga *et al*, (Zgaga *et al*, 2014), while many papers studied the impact on both overall survival or progression-free survival for example, Lohman *et al* (Lohmann *et al*, 2015). However, where multiple estimates were extracted, no patient was included more than once for a certain exposure or outcome. † Study authors were contacted to provide HR, RR or OR when not reported; 13 did not respond. ¥ One study (Vrieling *et al*, 2011) used the same breast cancer cohort as a later, larger study (Vrieling *et al*, 2014) and as both had the same NOS score, the newer study was included. 25OHD: 25-hydroxyvitamin D; DBP: vitamin D binding protein; HR: hazard ratio; PSA: prostate specific antigen; WOK: Web of Knowledge.

A flowchart illustrating study selection is shown in Figure 1. After removal of duplicates, the search yielded 3070 potential articles. Irrelevant articles were eliminated after screening titles (*N*=2708) or abstracts (*N*=262). One hundred full-texts were considered for inclusion and assessed for eligibility and 19 were excluded. Finally, 81 articles were kept for the systematic review and 64 of these were included in the meta-analysis. The main characteristics of included studies are summarised in Table 1 and Table 2.

### Assessment of included studies

**Figure 2:**
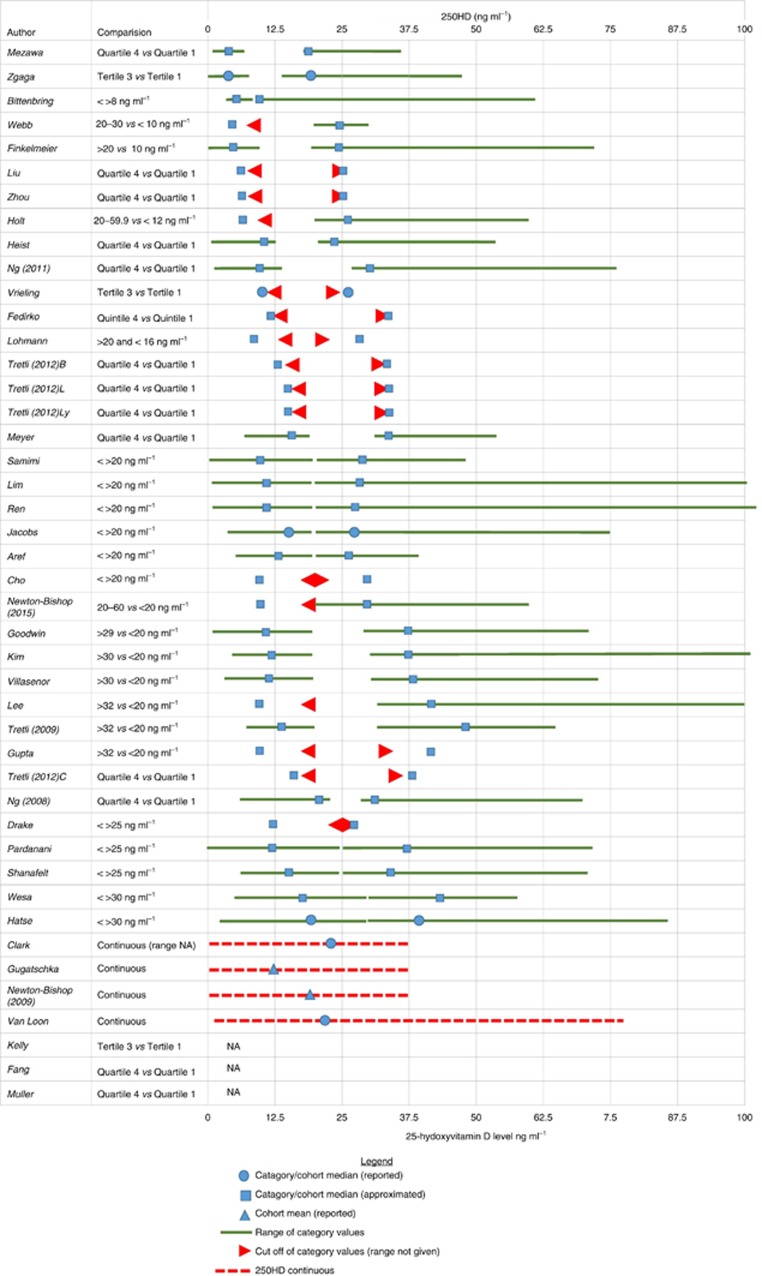
**Large variation in definition of vitamin D categories in studies included in systematic review.**Where not given in the paper, median 25OHD concentration for categories compared was requested from study authors and if not subsequently available was approximated. Approximation of the median for each category was performed using the cohort and/or category range where available. For categories defined by numerical cutoffs of 25OHD, the median for the lower category was approximated as the lowest reported 25OHD value (or 0 if category range not given) added to the midpoint of the category upper cutoff minus the lowest reported 25OHD value. For example, Bittenbring *et al* (Bittenbring *et al*, 2014) reported outcome according to 25OHD <>8 ng ml^−1^ groups and reported a study cohort range of 4–61.9. The median of the lower category (<8 ng ml^−1^) was approximated as the lowest value in the range plus the midpoint of the category that is, 4+((8−4)/2)=6. The upper category median was approximated as the category cutoff (that is, the lowest value in that category) added to the midpoint of the lower category. for example, in the Bittenbring *et al*, paper the median of the upper category was approximated as 8+((8−4)/2)=10. Where the compared categories were tertiles, quartiles or quintiles, the median of the lower category and upper categories was the midpoint of the difference between upper cutoff of the lower category compared and the lower cut-off of the higher category compared divided by the number of groups between two categories compared, either subtracted from the upper cutoff of the lower category or added to the lower cutoff of the higher category, respectively. For example, Bade *et al*, (Bade *et al*, 2014) grouped patients by quartile of 25OHD and report a cohort range of 4–59.6 ng ml^−1^. Q1 is given as 25OHD<9.86 ng ml^−1^ and Q4 >24.4 ng ml^−1^. Therefore, the medians of Q1 and Q4 were approximated as follows: Q1(median)=9.86−(((24.4−9.86)/2)/2)=6.225 and Q4(median)=24.4+(((24.4−9.86)/2)/2)=28. Insufficient data were reported in three studies to allow graphical illustration of categories or approximation of median. NA=data not reported; For Tretli *et al*, study: B=breast; C=colon; L=lung; Ly=lymphoma.

The risk of bias assessment revealed that 35 studies (43%) had a low risk of bias, 35 (43%) had an uncertain, and 11 (14%) had a high risk of bias. The risk of bias assessment summary per each domain is shown in Supplementary Figure S1 and individual study scores in Supplementary Figure S2. Sixty-four studies were included in the meta-analysis, with a total of 44 165 patients. Most studies were conducted in the USA (*N*=24) and Europe; breast cancer was most commonly studied (*N*=15), followed by nine studies (each) on prostate cancer and colorectal cancer. In total, 157 HR estimates for a range of exposure-outcome pairs were included in meta-analyses: 77 estimates (from 41 studies) for association with 25OHD, and 80 estimates (from 27 studies) relating to genetic factors. Separate estimates were extracted for different patient subgroups (e.g., different type of haematological malignancy (Drake *et al*, 2010)), different exposures (e.g., multiple polymorphisms (Zgaga *et al*, 2014)), or different outcome (i.e., survival or disease progression (Lohmann *et al*, 2015)). No patients were included more than once in meta-analysis, as separate meta-analyses have been conducted for each exposure-outcome pair. Very large differences were observed in definition of vitamin D categories being compared. For example, the median 25OHD concentration was 18.26 ng ml^−1^ in the ‘high' category in one study, (Zgaga *et al*, 2014) yet this was actually lower than the median (19.7 ng ml^−1^) in the ‘low' category in another study (Hatse *et al*, 2012). The variety of vitamin D categories, cutoffs and means/medians used are presented in Figure 2 and Supplementary Figure S3.

### Meta-analysis of 25OHD studies

#### Circulating vitamin D and survival

**Figure 3:**
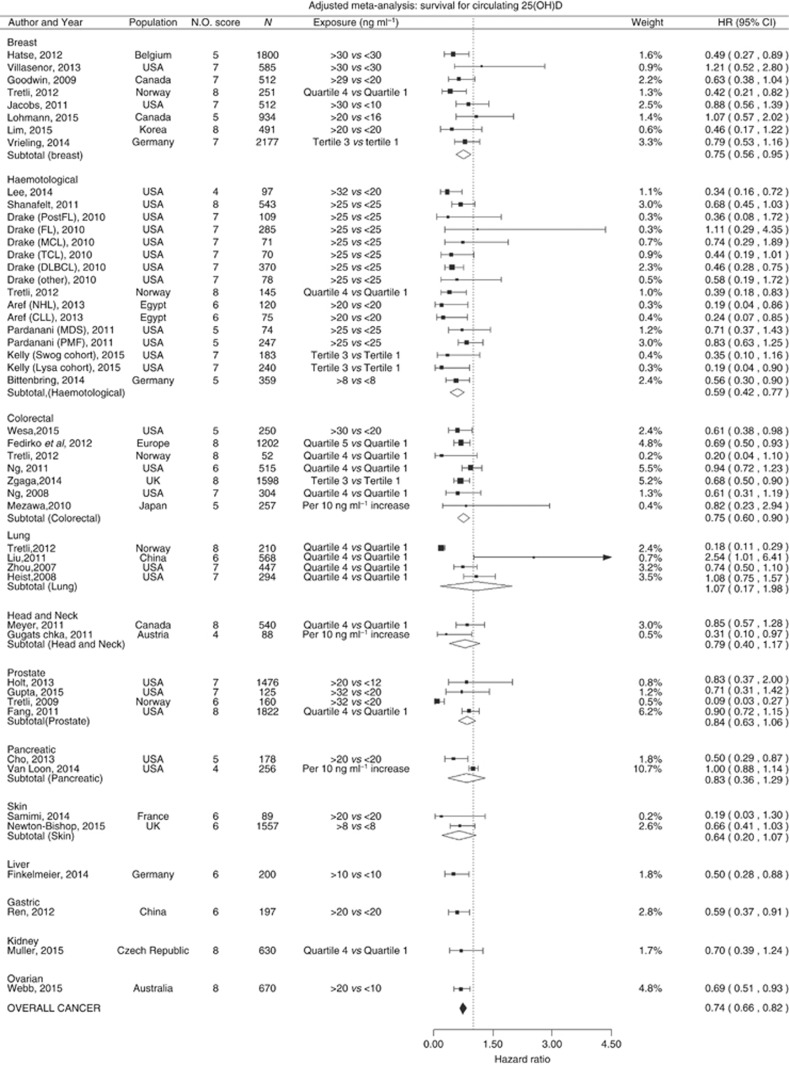
**Cancer survival and 25-hydroxyvitamin D concentration: meta-analysis of adjusted hazard ratios.**HR are sorted by cancer site and the difference in median between ‘high' and ‘low' vitamin D categories compared. Acute myeloid leukaemia (AML), Chronic Lymphoid Leukaemia (CLL), and subtypes of non- Hodgkin's lymphoma (NHL) (large B-cell lymphoma (DLBCL), T-cell lymphoma (TCL), Follicular Lymphoma (FL) and mantle cell lymphoma (MCL)) Myelodysplastic syndrome (MDS) and primary myelofibrosis (PMF). *I*^2^=breast: 0, haematological: 0, colorectal: 0.91, prostate: 0.68, head and neck: 0, pancreatic: 0.66, lung: 0.93, skin: 0, overall cancer: 0.18. Approximated Median in studies using quartiles/tertiles (ng ml^−1^): Tretli breast (lower: 12.9, upper: 33.9), Tretli Haematological: (lower:14.3, upper: 34.1), Tretli colorectal: (lower:16.4, upper: 38), Tretli lung: (lower:14.3, upper: 34.1), Vrieling: (lower: 10.6, upper: NA), Kelly (NA), Fedirko: (lower:11.8, upper: 33.4), Ng *et al* (2011): (lower:9.6, upper: 30.7), Zgaga: (lower:4.4, upper: 18.3), Ng *et al* (2008): (lower:21, upper: 30.6), Liu: (lower:7, upper: 25.4), Zhou: (lower:7.4, upper: 24.5), Heist: (lower:10.4, upper: 23.9), Meyer: (lower:16.2, upper: 34.2), Fang: (NA), Muller: (NA).

Forty-eight estimates from 38 studies were included in the meta-analysis of 25OHD and survival (17 studies (45%) examined cancer-specific mortality), comprising in total 24 013 cancer patients. Twelve cancer types were represented: breast, haematological, head and neck, colorectal, lung, prostate, skin, pancreatic liver, gastric, kidney and ovarian cancers. Overall, a significantly reduced risk of death was observed when comparing those with high to those with low vitamin D levels; meta-analysis HR=0.74, 95% CI=0.66 to 0.82 (Figure 3). The same significant trend was also observed in subgroup meta-analysis for breast (HR=0.75, 95% CI=0.56–0.95), haematological (HR=0.59, 95% CI=0.42–0.77) and colorectal cancers (HR=0.75, 95% CI=0.60–0.90). There was also a non-significant trend towards better survival with increased 25OHD observed in the subgroup analysis for prostate, skin, head and neck cancers. Virtually no change in direction or significance in overall effect was observed in sensitivity analyses when excluding studies at high risk of bias (HR=0.73, 95% CI=0.65–0.80), focusing on the studies at low risk of bias only (HR=0.72, 95% CI=0.63–0.81), excluding studies that used continuous 25OHD (HR=0.73, 95% CI=0.65–0.80), limited to cancer-specific mortality studies only (HR=0.75, 95% CI=0.65–0.84), or to studies that strictly reported HR (HR=0.74, 95% CI=0.66–0.82). The same was true after selection of studies where the difference in mean/median between high and low categories being compared was over or below 20 ng ml^−1^; (HR=0.70, 95% CI=0.60–0.81, and HR=0.71, 95% CI=0.55–0.87, respectively), or when stratifying by the lower category mean/median below or greater than 12.5 ng ml^−1^ (HR=0.76, 95% CI=0.64–0.88, and HR=0.61, 95% CI=0.47–0.75, respectively) (for sensitivity analysis please see supplementary material).

#### The relationship between circulating vitamin D level and disease progression

**Figure 4:**
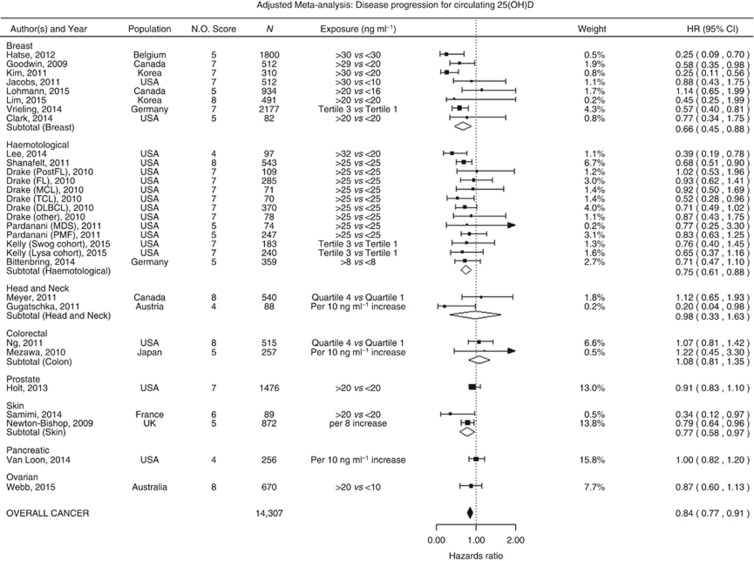
**Cancer progression and 25-hydroxyvitamin D concentration: meta-analysis of adjusted hazard ratios.**HR are sortd by the difference in median between high and low vitamin D levels compared. Acute myeloid leukaemia (AML), Chronic Lymphoid Leukaemia (CLL), and subtypes of non- Hodgkin's lymphoma (NHL) (large B-cell lymphoma (DLBCL), T-cell lymphoma (TCL), follicular lymphoma (FL) and mantle cell lymphoma (MCL)). I2=breast: 0, haematological: 0, colorectal: 0, head and neck: 0, skin: 0 overall cancer: 0. Approximated median in studies using quartiles/tertiles (ng ml^−1^): Vrieling: (lower: 10.6, upper: NA), Kelly (NA), Meyer: (lower:16.2, upper: 34.2), Ng *et al*, (2011): (lower:9.6, upper: 30.7).

Twenty-three studies investigated the association between circulating 25OHD and disease progression; from these studies 29 estimates were included in our meta-analysis comprising in total 14 307 patients with breast, haematological, head and neck, colorectal, prostate, skin, pancreatic, or ovarian cancer. Higher circulating vitamin D was associated with a significant reduction in disease progression for all cancers combined (HR=0.84, 95% CI=0.77–0.91; Figure 4); this was also observed in subgroup meta-analysis of breast (HR=0.66, 95% CI=0.45–0.88), haematological (HR=0.75, 95% CI=0.61–0.88) and skin cancer (HR=0.77, 95% CI=0.58–0.97). Findings remain fundamentally unchanged after exclusion of studies at high risk of bias (HR=0.82, 95% CI=0.74–0.90), limited to studies at low risk of bias only (HR=0.80, 95% CI=0.70–0.90) or excluding studies that used continuous 25OHD (HR=0.81, 95% CI=0.73–0.90), or limited to studies that strictly reported HR (HR=0.84, 95% CI=0.77–0.91). The same was true after selection of studies where the difference in mean/median between high and low categories being compared was over or below 20 ng ml^−1^; (HR=0.81, 95% CI=0.72–0.90, and HR=0.75, 95% CI=0.55–0.95, respectively), or when stratifying by the lower category mean/median below or greater than 12.5 ng ml^−1^ (HR=0.84, 95% CI=0.71–0.97, and HR=0.77, 95% CI=0.62–0.92, respectively) (for sensitivity analysis please see supplementary material.

### Vitamin-D-related genetic variation

#### VDR and other vitamin D pathway SNPs and survival

**Figure 5:**
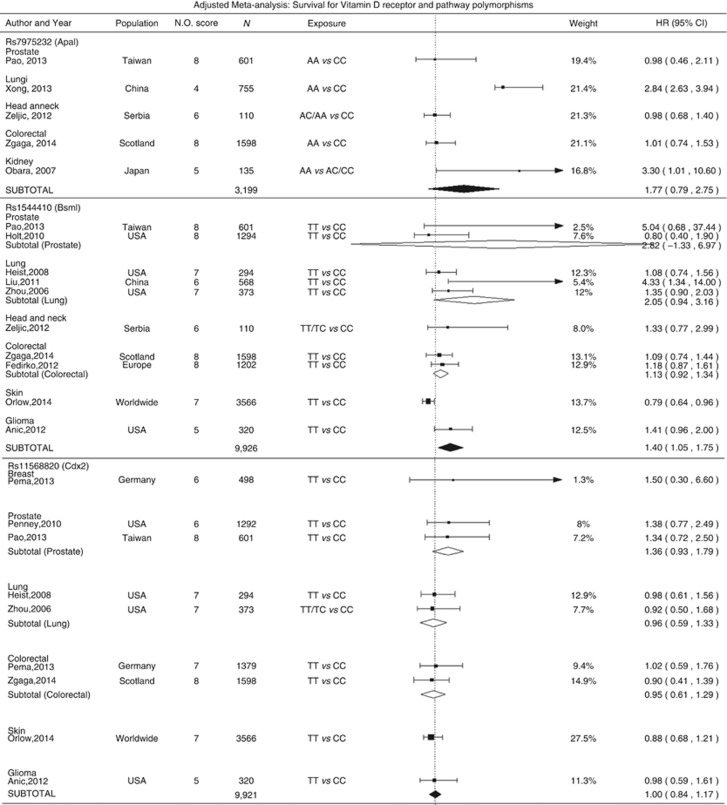

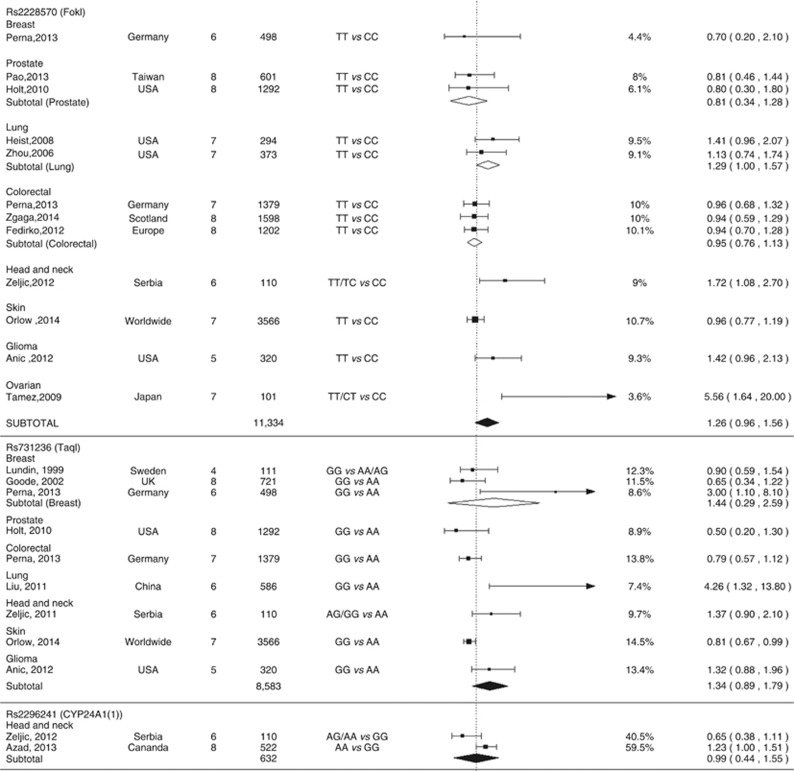

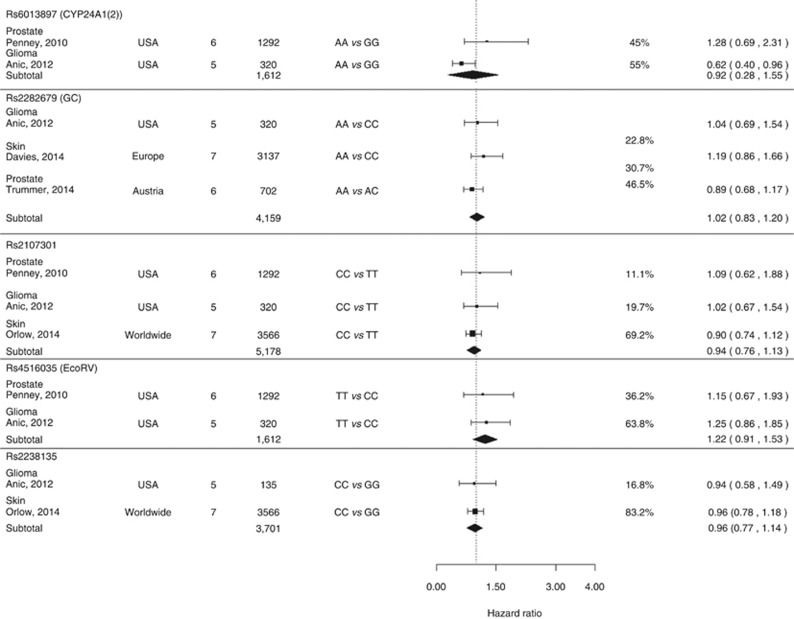
**Cancer survival and vitamin D receptor polymorphisms and other vitamin D-related genetic factors: adjusted meta-analysis.***I*^2^ for ApaI: 0.95, BsmI prostate: 0.93, BsmI Lung: 0.93, BsmI colorectal: 0, BsmI All: 0.85, Cdx2 prostate: 0, Cdx2 lung: 0, Cdx2 colorectal: 0, Cdx2 All: 0, FokI Prostate: 0, FokI lung: 0, FokI colorectal: 0, FokI All: 0.83, TaqI breast: 0.88, TaqI skin: 0.46, TaqI all: 0.86, Cyp24a1(1) all: 0.75, Cyp24a1(2) all: 0.67, GC all: 0, Rs2107301 all: 0, Rs4516035: 0, Rs2238135: 0.

Twenty-one studies investigated the association between vitamin-D-related genetic variation and survival; 10 (48%) examined cancer-specific mortality. By far, the most commonly studied were polymorphisms in *VDR* gene, particularly rs2228570 (*FokI),* rs1544410 (*BsmI),* rs731236 *(TaqI),* rs11568820 *(Cdx2)*, and *rs*7975232 (*ApaI)*. In meta-analysis, rs1544410 *TT/TC* genotypes were associated with worse survival compared to CC genotype (HR=1.40, 95% CI=1.05–1.75; Figure 5). The same direction of the effect was observed in the sensitivity analyses after exclusion of studies with NOS<7 (Supplementary Figure S4) and those reporting on cancer-specific mortality, but the association was no longer significant (Supplementary Figure S5). In lung cancer patients, a poorer outcome was observed to be associated with rs2228570 *TT/TC* carriers (HR=1.29, 95% CI=1.00–1.57) and a consistent albeit non-significant association was found across all cancers (HR=1.26, 95% CI=0.96–1.56). A significant association was observed with rs731236 *(Taq1)* variant when limited to studies at low risk of bias (NOS score ⩾7; HR=0.79, 95% CI=0.62–0.95, Supplementary Figure S4). Other genetic factors were investigated in at most three original studies and no other statistically significant results were observed.

#### VDR and vitamin D pathway SNPs and disease progression

**Figure 6:**
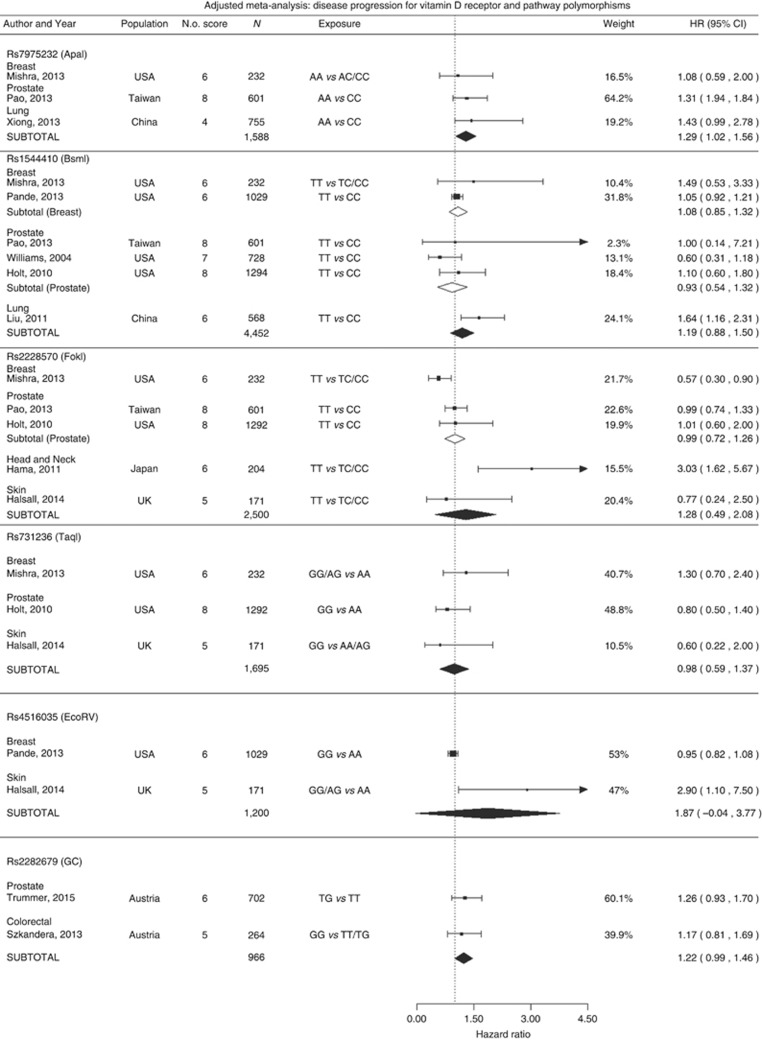
**Cancer progression and vitamin D receptor polymorphisms and other vitamin D-related genetic variants: adjusted meta-analysis.***I*^2^ for ApaI: 0, BsmI prostate: 0.52, BsmI breast: 0.1, BsmI All: 0.61, FokI Prostate: 0, FokI All: 0.90, TaqI all: 0, Rs4516035: 0.94, Rs22382679: 0.

Ten studies examined the effect of genetic variation on disease progression (Figure 6; for sensitivity analysis see Supplementary Figure S6). In meta-analysis of three studies with a total of 1588 patients, it was observed that *rs*7975232 *AA* carriers had significantly worse survival than CC carriers (HR=1.29, 95% CI=1.02–1.56). Additionally, a suggestive association was observed for vitamin D binding protein variant *rs2282679* (HR=1.22, 95% CI=0.99–1.46) in meta-analysis of two studies.

### Testing for publication bias and study heterogeneity

There was some evidence of heterogeneity between studies in meta-analysis of 25OHD and some evidence of publication bias (Supplementary Figures S7 and S8). A non-insignificant degree of heterogeneity and evidence of publication bias were observed in some subgroup analysis. Heterogeneity was observed for subgroup analysis of rs1544410, rs7975232, rs2228570 and rs731236, as well as for some individual cancer types while publication bias was observed for rs1544410, rs2228570 and rs731236 (Supplementary Figures S7 and S8).

### Studies not included in meta-analysis

Seventeen papers were excluded from the meta-analysis, but their findings were nonetheless considered (Table 2). Eight studies report improved overall and/or progression-free survival among those with higher 25OHD concentration (Vrieling *et al*, 2011; Walentowicz-Sadlecka *et al*, 2012; Peiris *et al*, 2013; Field *et al*, 2013; Bade *et al*, 2014; Der *et al*, 2014; Hansson *et al*, 2014; Obermannova *et al*, 2015) and one study found no association between 25OHD and incidence of metastases (Nurnberg *et al*, 2009). Seven studies investigated genetic variants and outcome (median sample size: 66). One study reported that the rs731236/rs2228570 (*TaqI-FokI, TTFf/TtFf)* haplotype was significantly associated with reduced overall survival (HR=1.81, 95% CI=1.23–3.48, *P*=0.04) (Turna *et al*, 2012): suggestive associations were reported between progression-free survival and rs731236 (*AA*) genotype in prostate cancer (Furuya *et al*, 1999) and rs2228570 *TT* genotype in breast cancer (Yiallourou *et al*, 2014), while there was no association found between rs2228570 and paediatric ALL (Kim *et al*, 2012). No association was observed between rs1544410 and breast cancer outcome (Yagmurdur *et al*, 2009). There was a suggestive association between platelet VDR expression and survival in ovarian cancer (Silvagno *et al*, 2010). Finally, low vitamin D binding protein (DBP) levels were found to be predictive of lung cancer death (Turner *et al*, 2013).

## Discussion

This is the first systematic review with meta-analysis that examines the relationship between cancer outcomes and variation in vitamin D pathway genes, and also by far the largest review on vitamin D status and cancer outcome. Our review suggests that higher circulating vitamin D in cancer patients is associated with a 26% lower rate of death and a 16% lower rate of disease progression. The clear association with survival was also observed in site-specific analyses of breast, haematological and colorectal cancers, while an association with reduction in disease progression was also found in those diagnosed with breast, haematological and skin cancer.

Establishing a causal relationship between vitamin D status and cancer progression is challenging because risk factors associated with cancer outcome are often also associated with vitamin D deficiency. For example, the association between 25OHD and improved survival observed in the original studies might be due to 25OHD being a marker of healthier lifestyle (i.e., healthier diet containing more fish; physical activity and spending time outdoors). However, evidence that genetic factors linked to vitamin D metabolism and pathways impact upon cancer survival may be used to counter such concerns and support a causal link. In our meta-analysis, we found evidence of an association between the *VDR* gene variants with functionally characterised effects and cancer outcome. Forty percent higher rate of death was observed in *TT* carriers at rs1544410 locus and 26% higher rate in *TT* carriers at rs2228570, while 29% increased risk of disease progression was observed in *AA* carriers at *rs*7975232 and 22% in *GG* carriers at GC locus.

Evidence from biological studies support a role for these polymorphisms in modulating vitamin D biology. For example, rs2228570 has been shown to affect the translational start site of 1,25(OH)_2_D and hence its downstream effects (Uitterlinden *et al*, 2004), while rs1544410 and *rs*7975232 have been associated with changes in *VDR* messenger RNA expression (Staal *et al*, 1996; Uitterlinden *et al*, 2004). We hypothesise that interactions between mutations in the vitamin D pathway and vitamin D status exist, and that this interaction could have a critical role in cancer prognosis. Indeed, Han *et al* (Han *et al*, 2007) have shown an interaction between vitamin D intake and rs1544410 polymorphism on cancer risk, and we and others have previously shown a modification of the relationship between vitamin D intake or status and cancer outcome by other *VDR* variants, thus suggesting an interaction of genetic and environmental factors (Li *et al*, 2007; Theodoratou *et al*, 2008; Anderson *et al*, 2011; Zgaga *et al*, 2014). In conjunction with the strong associations observed for vitamin D status, evidence from genetic studies further supports an important role of vitamin D in cancer progression.

Few studies to date have analysed the associations between *VDR* or vitamin D pathway genetic variants and cancer outcomes, and no meta-analyses have been published to date. A review by Kostner *et al* (Kostner *et al*, 2009) concluded that associations between *VDR* polymorphisms and cancer prognosis are strongest for prostate cancer (rs2228570), breast cancer (rs1544410, rs731236) malignant melanoma (rs1544410), and renal cell carcinoma (rs731236) but did not perform meta-analysis on these data.

Interestingly, Afzal *et al* (Afzal *et al*, 2014) have employed principles of Mendelian randomization in a study comprising 95 766 participants and found that variation in genes involved in vitamin D and 25OHD synthesis *(DHCR7* and *CYP2R1)* were associated with both all-cause and cancer mortality, supporting a causal role of vitamin D. To date, there are no published findings from randomised controlled trials (RCT) assessing the effect of vitamin D supplementation on survival in cancer patients, although several ongoing trials (unfortunately only some of which have disease progression as an outcome) were identified (ClinicalTrials.gov, 2016). Meanwhile, the data on cancer mortality from RCTs conducted in the general population can offer some insight; most notably, a Cochrane review of randomised studies comparing vitamin D supplements to placebo identified a significant reduction in cancer mortality in those taking vitamin D supplements (HR=0.88, 95% CI=0.78–0.98; Bjelakovic *et al*, 2014).

A major issue that is typically taken poor notice of in vitamin D meta-analyses—namely, a very large variability in vitamin D category definition amongst studies, is for the first time being highlighted and transparently shown in our review. Vitamin D categories differed in level as well as range—as a result, large heterogeneity in exposure definition occurred and study point-estimates are difficult to compare: it is, for example, unsurprising that the reported effect per 20 ng ml^−1^ is greater than effect per 5 ng ml^−1^ increase. Therefore, there is a need for a consensus in category definition and reporting of effect sizes: future original studies should report effect sizes using internationally agreed cutoffs, such as those given by the Institute of Medicine, solely or in addition to study-specific cutoff values chosen. Generally, variability in exposure categories results in a more heterogeneous estimates and is likely to increase statistical uncertainty and hence bias results towards the null. Nonetheless, our summary findings remain largely unchanged when the analysis was limited according to the difference in 25OHD between the compared groups.

There are some additional limitations of the present work. First, a number of relevant studies were published after the time limits stipulated in our search strategy and so are not included in our meta-analysis. Some such papers support the conclusions presented here (Brandstedt *et al*, 2016; Fang *et al*, 2016; Fanidi *et al*, 2016; Mondul *et al*, 2016; Orlow *et al*, 2016; Yao *et al*, 2016; Yuan *et al*, 2016), while others reported no association between circulating vitamin D and cancer outcome (Vashi *et al*, 2015; Ahn *et al*, 2016; Danilovic *et al*, 2016; McGovern *et al*, 2016).

Second, various assays were used for 25OHD measurement in the different studies, while 25OHD was also sampled at variable timepoints, including pre-diagnosis, before treatment and after treatment, which may impact the results. Also, in disease progression studies, different outcome definitions were used for example, disease-free survival, local or distant recurrence.

In the present study, results for all cancers combined are given, in addition to site-specific findings, we yet fully acknowledge that cancer is a heterogeneous disease. However, numerous studies have shown involvement of vitamin D on key hallmarks of cancer, many of which are common to all cancers; preclinical studies demonstrate effects on cell cycle arrest, cell adhesion, differentiation, proliferation, tumour angiogenesis, and apoptosis in human cancer cell lines (Simboli-Campbell *et al*, 1997; Chen *et al*, 2000; Krishnan *et al*, 2003; Deeb *et al*, 2007; Kizildag *et al*, 2010; Hsu *et al*, 2011; Ting *et al*, 2012), while reduction in cancer proliferation has been shown in carcinogen-exposed rats (Mokady *et al*, 2000) and cancer phenotypes are more commonly observed in vitamin D receptor (*VDR*) knockout mice (Zheng *et al*, 2012). Nevertheless, the heterogeneity in pooled results between different cancer types and the small number of studies for certain cancers limits the strength of the current study in demonstrating an association between circulating 25-hydroxyvitamin D and total cancer survival.

Next, in reporting the impact of genetic variation on outcome, we acknowledge that ethnic differences in VDR variation exist, which might interfere with the findings from genetic studies, as ethnicity is directly linked to the skin type and vitamin D synthesis. Meanwhile, VDR variants may interact with circulating 25OHD to impact outcome, yet only a small number of studies examined these putative gene–environment interactions. Finally, we observed some evidence of heterogeneity and publication bias overall; however, findings from sensitivity analysis were highly consistent and supportive of main findings.

Despite these limitations, the present work includes a novel meta-analysis, investigating the association between vitamin D-related genetic variation and cancer outcome, in addition to a ∼50% larger meta-analysis of circulating 25OHD and cancer outcome compared to a previous review (Li *et al*, 2014). Moreover, stringent quality assessment of original studies and corresponding sensitivity analysis were conducted and strikingly inconsistent 25OHD category definitions were addressed in stratified analysis.

In conclusion, the consistent evidence across the studies presented in the current review demonstrates a clear and strong association between low baseline vitamin D levels and poorer cancer survival. The associations between vitamin D-related genetic variants and cancer survival support an interpretation that vitamin D may play an important role in influencing cancer outcome. However, a causal link cannot be conclusively established from observational studies; hence, well-designed and adequately powered RCTs are needed to evaluate the clinical application of vitamin D in augmenting standard follow-up and adjuvant chemotherapy regimens. Understanding the mechanism of action of genetic factors promises to provide further insight into biological determinants of response to treatment and could help inform prognosis.

## Figures and Tables

**Table 1:** Characteristics of studies (*N*=64) included in the meta-analysis

								**Variable**		**Outcome**	
**First author, year**	**Cancer (subtype)**	**HR/OR**	**Sample Size**	**Site**	**Follow-up (m)**	**Events**	**NOS**	**25OHD**	**Genetic**	**Progression**	**Survival**
Anic *et al* (2012)	Brain (glioma)	HR	320	USA	28	248 cancer deaths	5		✓		CS
Lim *et al* (2015)	Breast	HR	491	Korea	86	32 recurrences; 22 cancer deaths	8	✓ BT		DFS	CS
Lohmann *et al* (2015)	Breast	HR	934	Canada	112	Not given	4	✓ BT		RFS	OS
Clark *et al* (2014)	Breast	HR	82	USA	>36	23 relapses or deaths	5	✓ BT		RFS	
Vrieling *et al* (2014)	Breast	HR	2177	Germany	64	206 cancer deaths, 241 recurrences or deaths	7	✓ 66% BT		DFS	CS
Mishra *et al* (2013)	Breast	OR	232	USA	NA	Not given	5		✓	DFS	
Pande *et al* (2013)	Breast	HR	1029	USA	114	266 recurrences or deaths	6		✓	DFS	
Perna *et al* (2013a)	Breast	HR	498	Germany	60	48 cancer deaths	7		✓		CS
Villasenor *et al* (2013)	Breast	HR	585	USA	110	48 cancer deaths	7	✓ AT			CS
Hatse *et al* (2012)	Breast	HR	1800	Belgium	56	118 relapses; 64 cancer deaths	5	✓ BT		DFI	CS
Jacobs *et al* (2011)	Breast	OR	512	USA	88	Not given	5	✓ AT		R	OS
Kim *et al* (2012)	Breast	HR	310	Korea	23	33 metastases or deaths	7	✓ BT		DFS	
Goodwin *et al* (2009)	Breast	HR	512	Canada	139	116 recurrences; 106 deaths	7	✓ BT		R	OS
Goode *et al* (2002)	Breast	HR	721	UK	NA	200 deaths	6		✓		OS
Lundin *et al* (1999)	Breast	RR	111	Sweden	67	44 deaths	4		✓		OS
Tretli *et al* (2012)	Breast, colon, lung, and lymphoma	HR	658	Norway	>60	343 cancer deaths	7	✓ BT			CS
Wesa *et al* (2015)	Colorectal	HR	250	USA	NA	153 deaths	5	✓ BT			OS
Zgaga *et al* (2014)	Colorectal	HR	1598	UK	107	363 cancer deaths	8	✓ AT	✓		CS
Perna *et al* (2013b)	Colorectal	HR	1397	Germany	60	336 cancer deaths	6		✓		CS
Szkandera *et al* (2013)	Colorectal	HR	264	Austria	53	45 recurrences	5		✓	R	
Fedirko *et al* (2012)	Colorectal	HR	1202	Europe	73	444 cancer deaths	8	✓ BD	✓		CS
Ng *et al* (2011)	Colorectal	HR	515	USA	61	440 progression; 475 deaths	5	✓ BT		TTP	OS
Mezawa *et al* (2010)	Colorectal	HR	257	Japan	32	30 cancer deaths; recurrences not given	5	✓ NS		DFS	CS
Ng *et al* (2008)	Colorectal	HR	304	USA	78	96 cancer deaths	7	✓ BD			CS
Ren *et al* (2012)	Gastric	HR	197	China	>60	106 deaths	5	✓ BT			OS
Lee *et al* (2014)	Haematological (AML)	HR	97	USA	16	55 relapses; 51 deaths	4	✓ BT		R	OS
Shanafelt *et al* (2011)	Haematological (CLL)	HR	543	USA	118	201 progression; 96 deaths	8	✓ NS		TTT	OS
Aref *et al* (2013)	Haematological (CLL, NHL)	HR	195	Egypt	60	118 deaths	5	✓ BT			OS
Drake *et al* (2010)	Haematological (DLBCL)	HR	983	USA	35	404 events; 168 cancer deaths	6	✓ 66% BT		EFF	CS
Pardanani *et al* (2011)	Haematological (PMF, MDS)	HR	321	USA	34	36 progression; 171 deaths	4	✓ BT		LFS	OS
Bittenbring *et al* (2014)	Haemotological (BCL)	HR	359	Germany	49	Not given	4	✓ AT		EFF	OS
Kelly *et al* (2015)	Haemotological (FL)	HR	423	USA	65	193 progression; 58 deaths	5	✓ BT		PFS	OS
Azad *et al* (2013)	Head and neck	HR	522	Canada	>53	214 deaths	8		✓		OS
Zeljic *et al* (2012)	Head and neck	OR	110	Serbia	28–100	Not given	5		✓		CS
Meyer *et al* (2011)	Head and neck	HR	540	Canada	96	119 recurrences; 223 deaths	8	✓ BT		R	OS
Gugatschka *et al* (2011)	Head and neck (SCC)	RR	88	Austria	NA	31 progression; 29 deaths	4	✓ BT		DFS	OS
Hama *et al* (2011)	Head and neck (SCC)	HR	204	Japan	34	103 progression or deaths	6		✓	DFS	
Finkelmeier *et al* (2014)	Liver (HCC)	HR	200	Germany	11	60 deaths	6	✓ BT			OS
Zhou *et al* (2007)	Lung	HR	447	USA	72	126 cancer deaths	7	✓ BT			CS
Liu *et al* (2011)	Lung (AC, SCC)	HR	568	China	19	311 deaths	6	✓ NS	✓		OS
Heist *et al* (2008)	Lung (AC, SCC)	HR	294	USA	42	233 deaths	6	✓ NS	✓		OS
Zhou *et al* (2006)	Lung (AC, SCC)	HR	373	USA	71	186 deaths	7		✓		OS
Xiong *et al* (2013)	Lung (NSCC)	HR	755	China	NA	Not given	4		✓	PFS	OS
Newton-Bishop *et al* (2015)	Melanoma	HR	2182	UK	NA	Not given	6	✓ NS			CS
Davies *et al* (2014)	Melanoma	HR	3137	Various	96	653 deaths	7		✓		OS
Orlow *et al* (2014)	Melanoma	HR	3566	World wide	91	254 cancer deaths	7		✓		CS
Newton-Bishop *et al* (2009)	Melanoma	HR	872	UK	56	173 relapses	5	✓ NS		DFS	
Halsall *et al* (2004)	Melanoma	HR	171	UK	75	18 metastases	4		✓	M	
Webb *et al* (2015)	Ovarian	HR	670	Australia	>60	491 progression; 435 deaths	7	✓ BT		PFS	OS
Tamez *et al* (2009)	Ovarian	HR	101	Japan	85	28 cancer deaths; total deaths not given	7		✓		OS
Van Loon *et al* (2014)	Pancreatic	HR	256	Europe	35	progression not given; 254 deaths	4	✓ BT		PFS	OS
Cho *et al* (2013)	Pancreatic	HR	178	USA	33	82 deaths	5	✓ BT			OS
Gupta *et al* (2015)	Prostate	HR	125	USA	31	49 deaths	7	✓ BT			OS
Trummer *et al* (2015)	Prostate	HR	702	Austria	73–91	93 metastases; 123 deaths	6		✓	M	OS
Holt *et al* (2013)	Prostate	HR	1476	USA	130	325 progression; 95 cancer deaths	7	✓ NS		P	CS
Pao *et al* (2013)	Prostate	HR	601	Taiwan	60–120	415 progression; 101 cancer deaths	8		✓	P	CS
Fang *et al* (2011)	Prostate	HR	1822	USA	120	166 cancer deaths	8	✓ BD			CS
Holt *et al* (2010)	Prostate	HR	1294	USA	102	139 recurrences; 57 cancer deaths	8		✓	R	CS
Penney *et al* (2010)	Prostate	OR	1292	USA	>60	Not given	5		✓		OS
Tretli *et al* (2009)	Prostate	HR	160	Norway	44	52 cancer deaths	6	✓ 77% BT			CS
Williams *et al* (2004)	Prostate	HR	728	USA	60–120	Not given	7		✓	DFS	
	Renal	HR	630	Europe	30	152 cancer deaths	8	✓ BT			CS
Obara *et al* (2007)	Renal (RCC)	RR	135	Japan	>60	Not given	5		✓		CS
Samimi *et al* (2014)	Skin (Merkel cell)	HR	89	France	NA	33 metastases; 19 deaths	6	✓ NS		M	CS

Abbreviations: AC=adenocarcinoma; ALL=acute lymphocytic leukaemia; AML=acute myeloid leukaemia; AT 25OHD=assayed after cancer treatment; BCL=B-cell lymphoma; BD 25OHD=assayed before diagnosis; BT 25OHD=assayed before treatment; CML=chronic myeloid leukaemia; CS=cancer-specific survival; DFI=disease-free interval; DFS=disease-free survival; DLBCL diffuse large B-cell lymphoma; EFF=event-free survival; FL=follicular lymphoma; HCC=Hepatocellular carcinoma; LFS=leaukaemia-free survival; m months; M=metastasis; MDS=myelodysplastic syndrome; NA=not available; NHL=Non-Hodgkins lymphoma; NOS=Newcastle-Ottawa score; NS=Timing of 25OHD not specified/variable; NSCC=non-small-cell lung carcinoma; OS=overall-survival; P=progression not otherwise specified; PFS=progression-free survival; PMF=primary myelofibrosis; R=recurrence or relapse not otherwise specified; RCC=renal cell carcinoma; RFS=relapse/recurrence-free survival; SCC=squamous cell carcinoma; TTP=time to progression; TTT=time to treatment.

**Table 2:** Characteristics of studies (*N*=17) included in the qualitative synthesis

						**Variable**	**Outcome**				
**First author, year**	**Cancer (subtype)**	**Size**	**Follow-up (m)**	**Events**	**NOS**	**25OHD**	**Genetic**	**Progression**	**Survival**	**Author conclusion**	**Reason excluded**
Obermannova *et al* (2015)	Colorectal	84	24	Not given	4	✓		PFS	OS	Consistently low 25OHD (always <16 ng/ml) associated with worse PFS and OS	Serial 25OHD
Turner *et al* (2013)	Lung (NSCC)	142	52	Not given	7				CS	Low serum DBP levels predicted lung cancer-specific death (*P*=0.04)	Only paper reporting DBP
Turna *et al* (2012)	Lung(NSCC)	62	NA	Not given	5		✓		OS	Haplotype analysis revealed rs731236 (*TaqI)*—rs2228570 (*FokI) TTFf/TtFf* haplotype associated with reduced OS (*P*=0.04)	No individual SNP HR
Bade *et al* (2014)	Melanoma	324	NA	Not given	6	✓			OS	Increased 25OHD (Q4 v Q1) associated with increased OS 195 months v 80 months (*P*=0.049)	No HR
Der *et al* (2014)	Prostate	16 535	60	4613 deaths	5	✓			OS	Vitamin D deficiency significantly associated with reduced survival (<0.001)	No HR
Dickinson *et al* (2010)	Haematological (CML)	228	NA	55 relapses; 84 deaths	5		✓	R	OS	No data provided on impact of *VDR* variants	No HR
Furuya *et al* (1999)	Prostate	66	NA	Not given	3		✓	PFS		*TaqI TT* genotype associated with shorter PFS (*P*=0.07)	No HR
Hansson *et al* (2014)	Haematological (AML, ALL, CML, MDS)	123	96	29 relapses; 31 deaths	6	✓		R	OS	25OHD <20 ng/ml associated with reduced OS (*P*=0.01) and increased relapse (*P*=0.03)	No HR
Kim *et al* (2012)	Haematological	100	105	12 relapses; 4 deaths	4		✓	EFS	OS	*VDR* rs2228570 *FokI* genotype did not impact survival in paediatric ALL	No HR
Nurnberg *et al* (2009)	Melanoma	205	NA	118 metastases	4	✓		M		25OHD >20 ng/l associated with increased time to distant metastatic disease (*P*=0.64)	No HR
Peiris *et al* (2013)	Bladder	4126	NA	2025 deaths	6	✓			OS	25OHD <20 ng/ml associated with reduced OS (X2=10.44; *P*=0.001)	No HR
Silvagno *et al* (2010)	Ovarian (Epithelial)	26	NA	Not given	2		✓		OS	Increased platelet VDR expression (>50 fMol) associated with increased OS (*P*=0.12)	No HR
Walentowicz-Sadlecka *et al* (2012)	Ovarian	72	60	45 deaths	6	✓			OS	25OHD <10 ng/ml associated with reduced OS (*P*<0.04)	No HR
Yagmurdur *et al* (2009)	Breast	56	60	5 recurrences	3		✓	R		*rs1544410 (BsmI)* genotype not associated with local recurrence or metastasis *P*>0.05	No HR
Yiallourou *et al* (2014)	Breast	87	60	Not given	3		✓	PFS	OS	*rs2228570 FokI ff* associated with reduced PFS 35 months *vs* >54 months (*P*=0.08)	No HR
Field *et al* (2013)	Melanoma	795	56	137 cancer deaths	4	✓			CS	8 ng/ml incremental increase in 25OHD associated with improved DFS (*P*=0.02) and MSS (*P*=0.05)	Duplicate patient cohort
Vrieling *et al* (2011)	Breast	1295	70	182 recurrence or metastases; 183 deaths	7	✓		DFS	OS	Low 25OHD significantly associated with worse DFS and OS	Duplicate patient cohort

Abbreviations: AML=acute myeloid leukaemia, ALL=acute lymphocytic leukaemia; CML=chronic myeloid leukaemia; CS=cancer-specific survival; DFS=disease-free survival; DBP=vitamin D binding protein; EFF=event-free survival; fMol=femtomol; HR=hazard ratio; m=months; M=metastasis; MDS=myelodysplastic syndrome; MSS=melanoma specific survival; NOS=Newcastle-Ottawa score; NSCC=non-small-cell lung carcinoma; OS=overall survival; PFS=progression-free survival; R=recurrence or relapse not otherwise specified; SNP=single nucleotide polymorphism.

## Box 1.

Conversion of continuous HR and 95% CI estimate to per 10 ng ml^−1^ HR estimates.

To achieve this, we raised the continuous HR (or ‘HR per 1 ng ml^−1^') to the power of 10 to get *[A]*, per 10 ng ml^−1^ HR (e.g., continuous HR, 0.89, hence per 10 ng ml^−1^ HR: 0.89^10=0.3118). In order to calculate the confidence intervals we first found the exp(s.e.(beta))=*[B]*; the standard errors (s.e.) were calculated using the formula below. We then calculated 1.96*(x−xbar)=*[C]*, where x was fixed at 10 ng ml^−1^ and xbar was the median of all cohort means from the rest of the studies included in the meta-analysis. We found this to be 23 ng ml^−1^, therefore 1.96*(10−23)=−25.872=[C]. [B] was then raised to the power of *[C],* to get *[D].* Finally, the HR per 10 ng ml^−1^ *[A]* was multiplied or divided by *[D]* in order to derive the upper and lower 95% confidence intervals. Therefore, the resulting HR was *A* and 95% CI: (A*D) to (*A/D*).

(Continuous HR)^10=[*A*]=per 10 ng/ml HR

exp(s.e.(beta))=[*B*]

1.96*(x−xbar)=[*C*]

*B*^*C*=[*D*]

A*D=lower 95% CI

*A*/*D*=upper 95% CI.
