# Supplementary material for: The impact of vitamin D pathway genetic variation and circulating 25-hydroxyvitamin D on cancer outcome: systematic review and meta-analysis
Source: Br J Cancer. 2017 Mar 16;116(8):1092–110. doi: 10.1038/bjc.2017.44 (PMC5396104; doi:10.1038/bjc.2017.44)
Supplement: Supplementary Figure S2 [file bjc201744x2.docx]

| **First Author** | **Selection** | **Comparability*** | **Outcome** | **Total** |
| --- | --- | --- | --- | --- |
| *Anic* | *** | * | * | **5** |
| *Aref* | ** | ** | ** | **6** |
| *Azad* | *** | ** | *** | **8** |
| *Bade* | ** | * | *** | **6** |
| *Bittenbring* | *** | ** |  | **5** |
| *Cho* | ** | * | ** | **5** |
| *Clark* | *** | * | * | **5** |
| *Davies* | *** | * | *** | **7** |
| *Der* | ** | * | ** | **5** |
| *Dickinson* | ** | ** | *** | **7** |
| *Drake* | *** | ** | ** | **7** |
| *Fang* | *** | ** | *** | **8** |
| *Fedirko* | *** | ** | *** | **8** |
| *Field* | ** | * | * | **4** |
| *Finkelmeier* | *** | ** | * | **6** |
| *Furuya* | ** |  | * | **3** |
| *Goode* | *** | ** | *** | **8** |
| *Goodwin* | *** | ** | ** | **7** |
| *Gugatschka* | ** |  | ** | **4** |
| *Gupta* | *** | ** | ** | **7** |
| *Halsall* | *** | * | * | **5** |
| *Hama* | *** | ** | * | **6** |
| *Hansson* | ** | ** | ** | **6** |
| *Hatse* | *** | * | * | **5** |
| *Heist* | *** | ** | ** | **7** |
| *Holt (2010)* | *** | ** | *** | **8** |
| *Holt (201***)* | *** | ** | ** | **7** |
| *Jacobs* | ** | ** | *** | **7** |
| *Kelly* | *** | ** | ** | **7** |
| *Kim (2011)* | *** | ** | ** | **7** |
| *Kim (2012)* | ** | * | ** | **5** |
| *Lee* | ** | * | * | **4** |
| *Lim* | *** | ** | *** | **8** |
| *Liu* | ** | ** | ** | **6** |
| *Lohmann* | ** | * | ** | **5** |
| *Lundin* | ** |  | ** | **4** |
| *Meyer* | *** | ** | *** | **8** |
| *Mezawa* | ** | ** | * | **5** |
| *Mishra* | *** | ** | * | **6** |
| *Muller* | *** | ** | *** | **8** |
| *Newton-Bishop (2009)* | *** | * | * | **5** |
| *Newton-Bishop (2014)* | ** | * | *** | **6** |
| *Ng (2011)* | ** | ** | ** | **6** |
| *Ng (2008)* | *** | ** | ** | **7** |
| *Nurnberg* | ** | * | * | **4** |
| *Obara* | *** | * | * | **5** |
| *Obermannova* | ** | ** | ** | **6** |
| *Orlow* | *** | * | *** | **7** |
| *Pande* | *** | * | *** | **7** |
| *Pao* | *** | ** | *** | **8** |
| *Pardanani* | *** | * | * | **5** |
| *Peiris* | *** | * | ** | **6** |
| *Penney* | ** | * | *** | **6** |
| *Perna (201***)* | *** | ** | * | **6** |
| *Perna (201***)* | ** | ** | *** | **7** |
| *Ren* | ** | ** | ** | **6** |
| *Samimi* | *** | * | ** | **6** |
| *Shanafelt* | *** | ** | *** | **8** |
| *Silvagno* | ** |  |  | **2** |
| *Szkandera* | *** | * | * | **5** |
| *Tamez* | ** | ** | *** | **7** |
| *Tretli (2009)* | *** | * | ** | **6** |
| *Tretli (2012)* | *** | ** | *** | **8** |
| *Trummer* | ** | * | *** | **6** |
| *Turna* | ** | ** | ** | **6** |
| *Turner* | ** | ** | *** | **7** |
| *Van Loon* | ** |  | ** | **4** |
| *Villasenor* | *** | ** | ** | **7** |
| *Vrieling (2011)* | *** | * | *** | **7** |
| *Vrieling (2014)* | *** | * | *** | **7** |
| *Walentowicz-Sadlecka* | *** |  | *** | **6** |
| *Web* | *** | ** | *** | **8** |
| *Wesa* | *** |  | ** | **5** |
| *Williams* | *** | ** | ** | **7** |
| *Xiong* | ** | ** |  | **4** |
| *Yagmurdur* | ** |  | * | **3** |
| *Yiallourou* | ** |  | * | **3** |
| *Zeljic* | *** | ** | * | **6** |
| *Zgaga* | *** | ** | *** | **8** |
| *Zhou (2006)* | ** | ** | *** | **7** |
| *Zhou (2007)* | ** | ** | *** | **7** |
