# Supplementary material for: The impact of vitamin D pathway genetic variation and circulating 25-hydroxyvitamin D on cancer outcome: systematic review and meta-analysis
Source: Br J Cancer. 2017 Mar 16;116(8):1092–110. doi: 10.1038/bjc.2017.44 (PMC5396104; doi:10.1038/bjc.2017.44)
Supplement: Supplementary Figure S3 [file bjc201744x3.docx]

| **Author** | **Comparison** | **Study Cohort(s)** | | | **Lower Category** | | | **Upper Category** | | |
| --- | --- | --- | --- | --- | --- | --- | --- | --- | --- | --- |
|  |  | **Range** | **Mean** | **Median** | **Range** | **Mean** | **Median†** | **Range** | **Mean** | **Median†** |
| *Aref* | <>20ng/ml | 6.6–31.0 | NA | 16.1;18.8 | 6.6-20 | NA | 13.3 | 20-31.0 | NA | 26.7 |
| *Bade* | Q4 V Q1 | 4-59.6 | 16.2 | 18 | 4-9.86 | NA | 6.2 | 24.4-59.6 | NA | 28.0 |
| *Bittenbring* | <>8ng/ml | 4-61.9 | NA | 9.2; 12.8 | 4-8 | NA | 6 | 8-61.9 | NA | 10 |
| *Cho* | <>20ng/ml | NA | 22 | NA | <20 | NA | 10 | >20 | NA | 30 |
| *Clark* | Continuous | NA | 22.7 | 23.1 | NA | NA | NA | NA | NA | NA |
| *Der* | NA | NA | NA | NA | NA | NA | NA | NA | NA | NA |
| *Drake DLBCL* | <>25ng/ml | NA | 24.7 | NA | <25 | NA | 12.5 | >25 | NA | 37.5 |
| *Drake FL* | <>25ng/ml | NA | 27 | NA | <25 | NA | 12.5 | >25 | NA | 37.5 |
| *Drake MCL* | <>25ng/ml | NA | 28.8 | NA | <25 | NA | 12.5 | >25 | NA | 37.5 |
| *Drake Other* | <>25ng/ml | NA | 28.2 | NA | <25 | NA | 12.5 | >25 | NA | 37.5 |
| *Drake PostFL* | <>25ng/ml | NA | 23.2 | NA | <25 | NA | 12.5 | >25 | NA | 37.5 |
| *Drake TCL* | <>25ng/ml | NA | 28.1 | NA | <25 | NA | 12.5 | >25 | NA | 37.5 |
| *Fang* | Q4 V Q1 | NA | NA | NA | NA | 16.19; 17.55 | NA | NA | 38.35; 42.78 | NA |
| *Fedirko* | Q4 V Q1 | NA | NA | NA | <14.52 | 11.4 | 11.8 | >30.72 | 39.72 | 33.4 |
| *Field* | per 8ng/ml | 2.88-48.6 | NA | 19.8 | NA | NA | NA | NA | NA | NA |
| *Finkelmeier* | >20 V <10ng/ml | 1-72 | 17 | NA | 1-10 | NA | 5.5 | 20-72 | NA | 24.5 |
| *Goodwin* | >29 V <20ng/ml | 3-70.8 | 23.2 | NA | 3-20 | NA | 11.5 | 29-70.8 | NA | 37.5 |
| *Gugatschka* | Continuous | NA | 12 | NA | NA | NA | NA | NA | NA | NA |
| *Gupta* | >32 V <20ng/ml | NA | NA | NA | <20 | NA | 10 | >32 | NA | 42 |
| *Hansson* | <>20ng/ml | 5.2-38.8 | NA | NA | 5.2-19.6 | 13.2 | 12.6 | 20-38.8 | 25.2 | 27.4 |
| *Hatse* | <>30ng/ml | 2.6–86.9 | 27.1 | 25.5 | 2.6-30 | 14.4 | 19.7* | 30-86.9 | 40.5 | 38.6* |
| *Heist* | Q4 V Q1 | 1.0-55.5 | 20.6 | 20.25 | 1-12.6 | NA | 10.4 | 21.6-55.5 | NA | 23.9 |
| *Holt* | 20-59.9 V <12ng/ml | NA | NA | NA | <12 | NA | 6 | 20-59.9 | NA | 26 |
| *Jacobs* | <>20ng/ml | NA | 24.3 | NA | 4-19 | NA | 15* | 20-75 | NA | 27* |
| *Kelly* | T3 V T1 | 7.60-63.00 | NA | 31.0; 17 | NA | NA | NA | NA | NA | NA |
| *Kim* | >30 V <20ng/ml | 4.8–109 | 31.4 | NA | 4.8-20 | NA | 12.4 | 30-109 | NA | 37.6 |
| *Lee* | >32 V <20ng/ml | NA | NA | NA | <20 | NA | 10 | 32-100 | NA | 42 |
| *Lim* | <>20ng/ml | 2.6-100.8 | 33.53 | NA | 2.6-20 | 12.96 | 11.3 | 20-100.8 | 40.44 | 28.7 |
| *Liu* | Q4 V Q1 | NA | NA | NA | <10.1 | NA | 7.0 | >22.6 | NA | 25.4 |
| *Lohmann* | >20 V <16ng/ml | NA | 27.9 | NA | <16 | NA | 8 | >20 | NA | 28 |
| *Meyer* | Q4 V Q1 | 7.6-54 | NA | NA | 7.6-19.2 | 14.8 | 16.2 | 31.2-54 | 36.4 | 34.2 |
| *Mezawa* | Q4 V Q1 | 3-36 | NA | 10 | 3-7 | 5.2 | 4.8 | 16-36 | 21.9 | 18.3 |
| *Muller* | Q4 V Q1 | 3-53.6 | NA | 17.2 | NA | NA | NA | NA | NA | NA |
| *Newton-Bishop (2009)* | Continuous | NA | 18.42 | NA | NA | NA | NA | NA | NA | NA |
| *Newton-Bishop (2015)* | 20-60 V <20ng/ml | NA | NA | NA | <20 | NA | 10 | 20-60 | NA | 30 |
| *Ng (2008)* | Q4 V Q1 | 6.0-70 | NA | NA | 6-22.6 | 16.5 | 21 | 29-70 | 40 | 30.6 |
| *Ng (2011)* | Q4 V Q1 | 2.3-75.4 | 21 | 20 | 2.3-13.1 | NA | 9.6 | 27.2-75.4 | NA | 30.7 |
| *Nurnberg* | >20 V <10ng/ml | NA | 16.93 | 14.3 | <10 | NA | 5 | >20 | NA | 25 |
| *Obermannova* | <>16ng/ml | NA | NA | 12.16 | <16 | NA | 8 | >16 | NA | 24 |
| *Pardanani* | <>25ng/ml | 0-72 | NA | 25; 30 | 0-25 | NA | 12.5 | 25-72 | NA | 37.5 |
| *Peiris* | NA | 4.3-107 | 24.5 | NA | NA | NA | NA | NA | NA | NA |
| *Ren* | <>20ng/ml | 3-104 | 19.94 | NA | 3-20 | NA | 11.5 | 20-104 | NA | 28.5 |
| *Samimi* | <>20ng/ml | 1-48 | NA | 16 | 1-20 | NA | 10.5 | 20-48 | NA | 29.5 |
| *Shanafelt* | <>25ng/ml | 6.0-71.0 | 30.6; 26.6 | NA | 6-25 | NA | 15.5 | 25-71 | NA | 34.5 |
| *Tretli (2009)* | >32 V <20ng/ml | 7.6-64.8 | 28.8 | NA | 7.6-20 | NA | 13.8 | 32-64.8 | NA | 38.2 |
| *Tretli (2012)B* | Q4 V Q1 | NA | NA | NA | <16.4 | NA | 12.9 | >30.4 | NA | 33.9 |
| *Tretli (2012)C* | Q4 V Q1 | NA | NA | NA | <20 | NA | 16.4 | >34.4 | NA | 38 |
| *Tretli (2012)L* | Q4 V Q1 | NA | NA | NA | <17.6 | NA | 14.3 | >30.8 | NA | 34.1 |
| *Tretli (2012)Ly* | Q4 V Q1 | NA | NA | NA | <17.6 | NA | 14.3 | >30.8 | NA | 34.1 |
| *Van Loon* | Continuous | 4.0-77 | NA | 21.7 | NA | NA | NA | NA | NA | NA |
| *Villasenor* | >30 V <20ng/ml | 3.9-71.7 | NA | NA | 3.9-20 | 13.9 | 12.0 | 30-71.7 | 36.6 | 38.05 |
| *Vrieling (2011)* | >22 V <14ng/ml | 3.88- 96.15 | NA | 17.96 | 3.88-14 | NA | 10.3* | 22-96.15 | NA | 28.52* |
| *Vrieling (2014)* | T3 V T1 | NA | NA | NA | <14 | NA | 10.6* | >22 | NA | NR |
| *Walentowicz-Sadlecka* | <>10ng/ml | NA | 12.5 | NA | <10 | NA | 5 | >10 | NA | 15 |
| *Webb* | 20-30 V <10ng/ml | NA | 17.6 | NA | <10 | NA | 5 | 20-30 | NA | 25 |
| *Wesa* | <>30ng/ml | 5.4-57.6 | NA | 21 | 5.4-30 | NA | 17.7 | 30-57.6 | NA | 42.3 |
| *Zgaga* | T3 V T1 | 0-48.8 | 11.4 | 10.0 | 0-7.25 | 3.8 | 4.4* | 13.3-48.8 | 20.34 | 18.29* |
| *Zhou* | Q4 V Q1 | NA | NA | NA | <10.2 | 6 | 7.4 | >21.6 | 28.2 | 24.5 |
