# Supplementary material for: The impact of vitamin D pathway genetic variation and circulating 25-hydroxyvitamin D on cancer outcome: systematic review and meta-analysis
Source: Br J Cancer. 2017 Mar 16;116(8):1092–110. doi: 10.1038/bjc.2017.44 (PMC5396104; doi:10.1038/bjc.2017.44)
Supplement: Supplementary Figure S7 [file bjc201744x7.docx]

**S7a Figure**

S7b Figure

S7c Figure

**S7d Figure**
