# Supplementary material for: The impact of vitamin D pathway genetic variation and circulating 25-hydroxyvitamin D on cancer outcome: systematic review and meta-analysis
Source: Br J Cancer. 2017 Mar 16;116(8):1092–110. doi: 10.1038/bjc.2017.44 (PMC5396104; doi:10.1038/bjc.2017.44)
Supplement: Supplementary Figure S8 [file bjc201744x8.docx]

| **Disease progression 25(OH)D** | | | | | |
| --- | --- | --- | --- | --- | --- |
|  | **I^2** | **Q-Val** | **P-Val** | **Df** | **eggars reg test** |
| Breast | 0 | 5.34 | 0.62 | 7 | p= 0.77 |
| Haematological | 0 | 3.82 | 0.97 | 11 | p = 0.975 |
| Colon | 0 | 0.08 | 0.77 | 1 | NA |
| Head and Neck | 0 | 0.112 | 0.738 | 1 | NA |
| Skin | 0 | 0.68 | 0.41 | 1 | NA |
| All | 0 | 22.9 | 0.736 | 28 | 0.054 |
| **Survival 25(OH)D** | | | | | |
|  | **I^2** | **Q-Val** | **P-Val** | **Df** | **eggars reg test** |
| Breast | 0 | 4.7 | 0.69 | 7 | p= 0.85 |
| Haematological | 0 | 4.4 | 0.99 | 14 | p= 0.33 |
| Colorectal | 0.9188 | 3.22 | 0.78 | 6 | p= 0.43 |
| Prostate | 0.6805 | 2.15 | 0.54 | 3 | p= 0.23 |
| Head and Neck | 0 | 0.76 | 0.38 | 1 | NA |
| Pancreatic | 0.6634 | 2.97 | 0.08 | 1 | NA |
| Lung | 0.929 | 22.13 | 0.001 | 3 | p = 0.04 |
| Skin | 0 | 0.22 | 0.64 | 1 | NA |
| Overall | 0.184 | 57.34 | 0.1433 | 47 | p = .0004 |
| **Survival (VDR)** | | | | | |
|  | **I^2** | **Q-Val** | **P-Val** | **Df** | **eggars reg test** |
| rs7975232 (***ApaI)*** |  |  |  |  |  |
| Subtotal | 0.952 | 83.13 | 0.0001 | 4 | p = 0.42 |
| rs1544410 (***BsmI)*** |  |  |  |  |  |
| Prostate | 0.931 | 14.48 | 0.001 | 1 | NA |
| Lung | 0.9255 | 26.83 | 0.001 | 2 | P= 0.001 |
| Colorectal | 0 | 0.17 | 0.679 | 1 | NA |
| subtotal | 0.848 | 59.16 | 0.001 | 9 | p = 0.001 |
| rs11568820 (**Cdx2)** |  |  |  |  |  |
| Prostate | 0 | 0.0087 | 0.93 | 1 | NA |
| Lung | 0 | 0.02 | 0.87 | 1 | NA |
| Colorectal | 0 | 0.108 | 0.74 | 1 | NA |
| Subtotal | 0 | 3.98 | 0.86 | 8 | p = 0.1612 |
| rs10735810 (***FokI)*** |  |  |  |  |  |
| Prostate | 0 | 0.0007 | 0.97 | 1 | NA |
| Lung | 0 | 0.902 | 0.34 | 1 | NA |
| Colorectal | 0 | 0.011 | 0.995 | 2 | P= 0.94 |
| Subtotal | 0.83 | 65.1 | 0.0001 | 11 | p = 0.001 |
| ***TaqI*** |  |  |  |  |  |
| Breast | 0.8791 | 16.54 | 0.0003 | 2 | p = 0.005 |
| Skin | 0.4608 | 3.71 | 0.16 | 2 | 0.092 |
| Subtotal | 0.8559 | 55.52 | 0.0001 | 8 | p = 0.0016 |
| **CYP24A1(1)** |  |  |  |  |  |
| Subtotal | 0.746 | 3.93 | 0.047 | 1 | NA |
| **CYP24A1(2)** |  |  |  |  |  |
| Subtotal | 0.6717 | 3.0462 | 0.08 | 1 | NA |
| **GC** |  |  |  |  |  |
| Subtotal | 0 | 1.83 | 0.4 | 2 | p= 0.39 |
| **RS2107301** |  |  |  |  |  |
| Subtotal | 0 | 0.545 | 0.76 | 2 | p= 0.46 |
| **Rs4516035** |  |  |  |  |  |
| Subtotal | 0 | 0.093 | 0.76 | 1 | NA |
| **Rs2238135** |  |  |  |  |  |
| Subtotal | 0 | 0.005 | 0.94 | 1 | NA |
| ***Disease progression (VDR)*** | | | | | |
|  | ***I^2*** | ***Q-Val*** | ***P-Val*** | ***Df*** | ***eggars reg test*** |
| rs7975232 (***ApaI)*** |  |  |  |  |  |
| Subtotal | 0 | 0.632 | 0.73 | 1 | NA |
| rs1544410 (***BsmI)*** |  |  |  |  |  |
| Prostate | 0.5229 | 1.38 | 0.96 | 2 | 0.85 |
| Breast | 0.1034 | 1.12 | 0.29 | 1 | NA |
| subtotal | 0.612 | 12.88 | 0.02 | 5 | p = 0.81 |
| rs10735810 (***FokI)*** |  |  |  |  |  |
| Prostate | 0 | 0.002 | 0.96 | 1 | NA |
| Subtotal | 0.906 | 42.4 | 0.001 | 4 | P=0.98 |
| ***TaqI*** |  |  |  |  |  |
| Subtotal | 0 | 1.83 | 0.4 | 2 | p = 0.61 |
| **Rs4516035** |  |  |  |  |  |
| Subtotal | 0.9371 | 15.88 | 0.001 | 1 | NA |
| **Rs2282679** |  |  |  |  |  |
| Subtotal | 0 | 0.14 | 0.71 | 1 | NA |
