# Supplementary material for: The impact of vitamin D pathway genetic variation and circulating 25-hydroxyvitamin D on cancer outcome: systematic review and meta-analysis
Source: Br J Cancer. 2017 Mar 16;116(8):1092–110. doi: 10.1038/bjc.2017.44 (PMC5396104; doi:10.1038/bjc.2017.44)
Supplement: Supplementary file 5 [file bjc201744x1.docx]

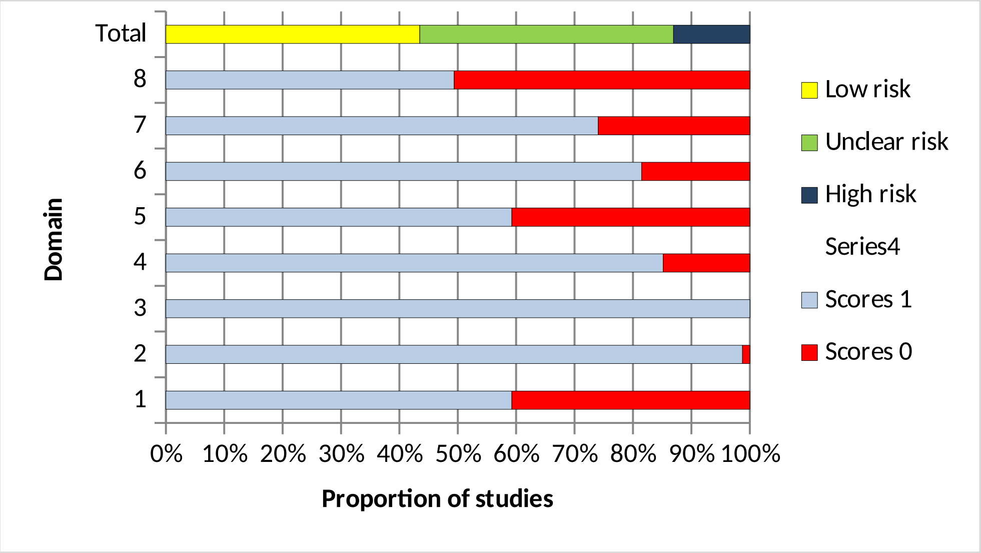
Total
8
7
6
5
4
3
2
1
Domain
0%
10%
20%
30%
40%
50%
60%
70%
80%
90%
100%
Proportion of studies
Low risk
Unclear risk
High risk
Series4
Scores 1
Scores 0
