# Supplementary material for: The impact of vitamin D pathway genetic variation and circulating 25-hydroxyvitamin D on cancer outcome: systematic review and meta-analysis
Source: Br J Cancer. 2017 Mar 16;116(8):1092–110. doi: 10.1038/bjc.2017.44 (PMC5396104; doi:10.1038/bjc.2017.44)

Adjusted Meta-analysis: Survival for Vitamin D receptor and pathway polymorphisms

Author and Year	Population	N.O. score	N	Exposure	Weight	HR [95% CI]
**Rs7975232 (ApaI)**						
**Prostate**						
Pao,2013	Taiwan	8	601	AA vs CC	*22.5%*	0.98 [0.46 , 2.11]
**Colorectal**						
Zgaga,2014	Scotland	8	1598	AA vs CC	*77.5%*	1.01 [0.74 , 1.53]
**SUBTOTAL**						**1.00 [0.64 , 1.37]**
**Rs1544410 (BsmI)**						
**Prostate**						
Pao,2013	Taiwan	8	601	TT vs CC	*2.0%*	5.04 [0.68 , 37.44]
Holt,2010	USA	8	1294	TT vs CC	*7.8%*	0.80 [0.40 , 1.90]
Subtotal (Prostate)						2.82 [-1.33 , 6.97]
**Lung**						
Heist,2008	USA	7	294	TT vs CC	*16.8%*	1.08 [0.74 , 1.56]
Zhou,2006	USA	7	373	TT vs CC	*15.9%*	1.35 [0.90 , 2.03]
Subtotal (Lung)						1.20 [0.93 , 1.47]
**Colorectal**						
Zgaga,2014	Scotland	8	1598	TT vs CC		1.09 [0.74 , 1.44]
Fedirko,2012	Denmark	8	1202	TT vs CC	*[illegible]*	1.18 [0.87 , 1.61]
Subtotal (Colorectal)						1.13 [0.92 , 1.34]
**Skin**						
Orlow ,2014	Worldwide	7	3566	TT vs CC	*20.6%*	0.79 [0.64 , 0.96]
**SUBTOTAL**						**1.14 [0.84 , 1.44]**
**Rs11568820 (Cdx2)**						
**Prostate**						
Pao,2013	Taiwan	8	601	TT vs CC	*9%*	1.34 [0.72 , 2.50]
**Lung**						
Heist,2008	USA	7	294	TT vs CC	*16.2%*	0.98 [0.61 , 1.56]
**Colorectal**						
Zhou,2006	USA	7	373	TT/TC vs CC	*9.7%*	0.92 [0.50 , 1.68]
Perna,2013	Germany	7	1379	TT vs CC	*11.8%*	1.02 [0.59 , 1.76]
Zgaga,2014	Scotland	8	1598	TT vs CC	*18.7%*	0.90 [0.41 , 1.39]
Subtotal (Colorectal)						0.95 [0.61 , 1.29]
**Skin**						
Orlow,2014	Worldwide	7	3566	TT vs CC	*34.6%*	0.88 [0.68 , 1.21]
**SUBTOTAL**						**0.96 [0.77 , 1.15]**
**Rs2228570 (FokI)**						
**Prostate**						
Pao,2013	Taiwan	8	601	TT vs CC	*10.4%*	0.81 [0.46 , 1.44]
Holt,2010	USA	8	1292	TT v CC	*8%*	0.80 [0.30 , 1.80]
Subtotal (Prostate)						0.81 [0.34 , 1.28]
**Lung**						
Heist,2008	USA	7	294	TT vs CC	*12.3%*	1.41 [0.96 , 2.07]
Zhou,2006	USA	7	373	TT vs CC	*11.8%*	1.13 [0.74 , 1.74]
Subtotal (Lung)						1.29 [1.00 , 1.57]
**Colorectal**						
Perna,2013	Germany	7	1379	TT vs CC	*12.9%*	0.96 [0.68 , 1.32]
Zgaga,2014	Scotland	8	1598	TT vs CC	*13%*	0.94 [0.59 , 1.29]
Fedirko,2012	Denmark	8	1202	TT vs CC	*13%*	0.94 [0.70 , 1.28]
Subtotal (Colorectal)						0.95 [0.76 , 1.13]
**Skin**						
Orlow ,2014	Worldwide	7	3566	TT vs CC	*13.8%*	0.96 [0.77 , 1.19]
**Ovarian**						
Tamez,2009	Japan	7	101	TT/CT vs CC	*4.8%*	5.56 [1.64 , 20.00]
**SUBTOTAL**						**1.22 [0.88 , 1.57]**

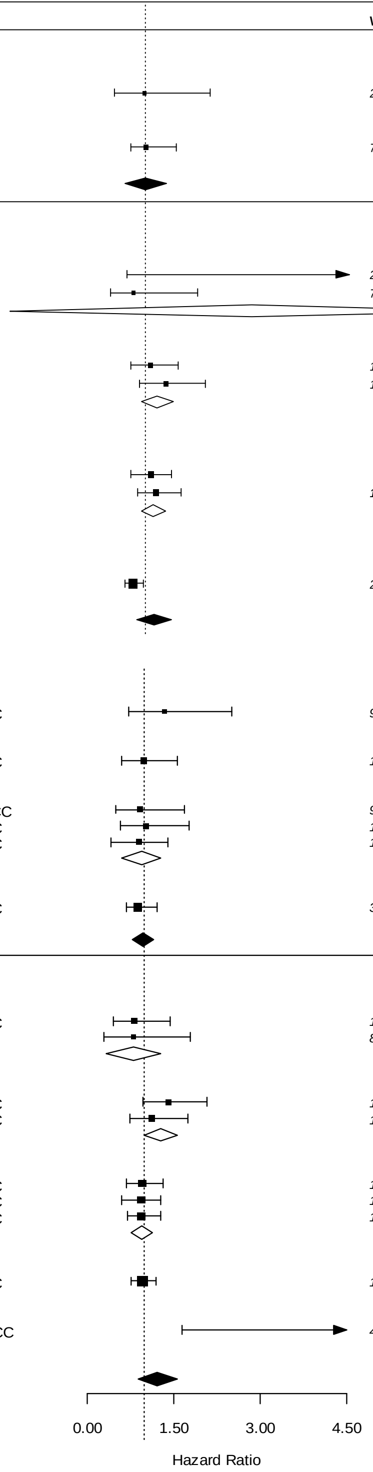

Adjusted Meta-analysis: Survival for Vitamin D receptor and pathway polymorphisms (continued)

Author and Year	Population	N.O. score	N	Exposure	Weight	HR [95% CI]
**Rs731236 (TaqI)**						
**Breast**						
Goode, 2002	UK	8	721	GG vs AA	*6.9%*	0.65 [0.34 , 1.22]
**Prostate**						
Holt, 2010	USA	8	1292	GG vs AA	*3%*	0.50 [0.20 , 1.30]
**Colorectal**						
Perna, 2013	Germany	7	1379	GG vs AA	*22.4%*	0.79 [0.57 , 1.12]
**Skin**						
Orlow, 2014	Worldwide	7	3566	GG vs AA	*67.7%*	0.81 [0.67 , 0.99]
**Subtotal**						**0.79 [0.62 , 0.95]**
**Rs2296241 (CYP24A1(1))**						
**Head and neck**						
Azad, 2013	Canada	8	522	AA vs GG	*100%*	1.23 [1.00 , 1.51]
**Rs2282679 (GC)**						
**Skin**						
Davies, 2014	Europe	7	3137	AA vs CC	*100%*	1.19 [0.86 , 1.66]
**Rs2107301**						
**Skin**						
Orlow, 2014	Worldwide	7	3566	CC vs TT	*100%*	0.90 [0.74 , 1.12]
**Rs2238135**						
**Skin**						
Orlow, 2014	Worldwide	7	3566	CC vs GG	*100%*	0.96 [0.78 , 1.18]

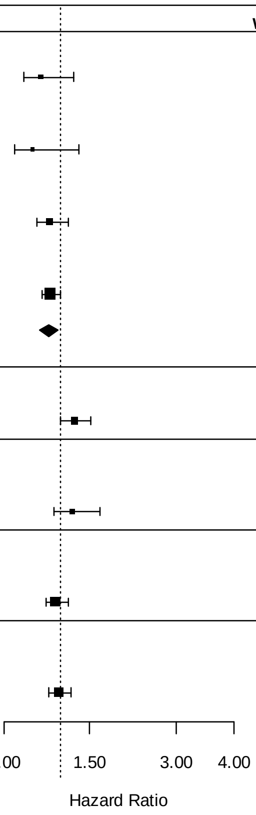

Supplement: Supplementary file 6 [file bjc201744x4.docx]
