# Supplementary material for: The impact of vitamin D pathway genetic variation and circulating 25-hydroxyvitamin D on cancer outcome: systematic review and meta-analysis
Source: Br J Cancer. 2017 Mar 16;116(8):1092–110. doi: 10.1038/bjc.2017.44 (PMC5396104; doi:10.1038/bjc.2017.44)

Adjusted Meta-analysis: Survival for Vitamin D receptor and pathway polymorphisms

Author and Year	Population	N.O. score	N	Exposure	Weight	HR [95% CI]
**Rs7975232 (Apal)**						
**Head and neck**						
Zeljic ,2012	Serbia	6	110	AC/AA vs CC	*22.8%*	0.98 [0.68 , 1.40]
**Colorectal**						
Zgaga,2014	Scotland	8	1598	AA vs CC	*38.2%*	1.01 [0.74 , 1.53]
**Kidney**						
Obara,2007	Japan	5	135	AA vs AC/CC	*39.0%*	3.30 [1.01 , 10.60]
**SUBTOTAL**						**1.52 [0.70 , 2.34]**
**Rs1544410 (Bsml)**						
**Prostate**						
Holt,2010	USA	8	1294	TT vs CC	*4.3%*	0.80 [0.40 , 1.90]
**Lung**						
Heist,2008	USA	7	294	TT vs CC	*14.8%*	1.08 [0.74 , 1.56]
**Head and neck**						
Zeljic,2012	Serbia	6	110	TT/TC vs CC	*4.8%*	1.33 [0.77 , 2.99]
**Colorectal**						
Zgaga,2014	Scotland	8	1598	TT vs CC	*19.1%*	1.09 [0.74 , 1.44]
Fedirko,2012	Denmark	8	1202	TT vs CC	*17.4%*	1.18 [0.87 , 1.61]
Subtotal (Colorectal)						1.13 [0.92 , 1.34]
**Skin**						
Orlow ,2014	Worldwide	7	3566	TT vs CC	*23.8%*	0.79 [0.64 , 0.96]
**Glioma**						
Anic,2012	USA	5	320	TT vs CC	*15.6%*	1.41 [0.96 , 2.00]
**SUBTOTAL**						**1.08 [0.89 , 1.28]**

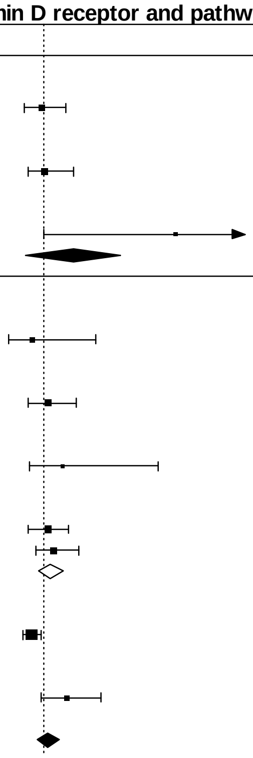

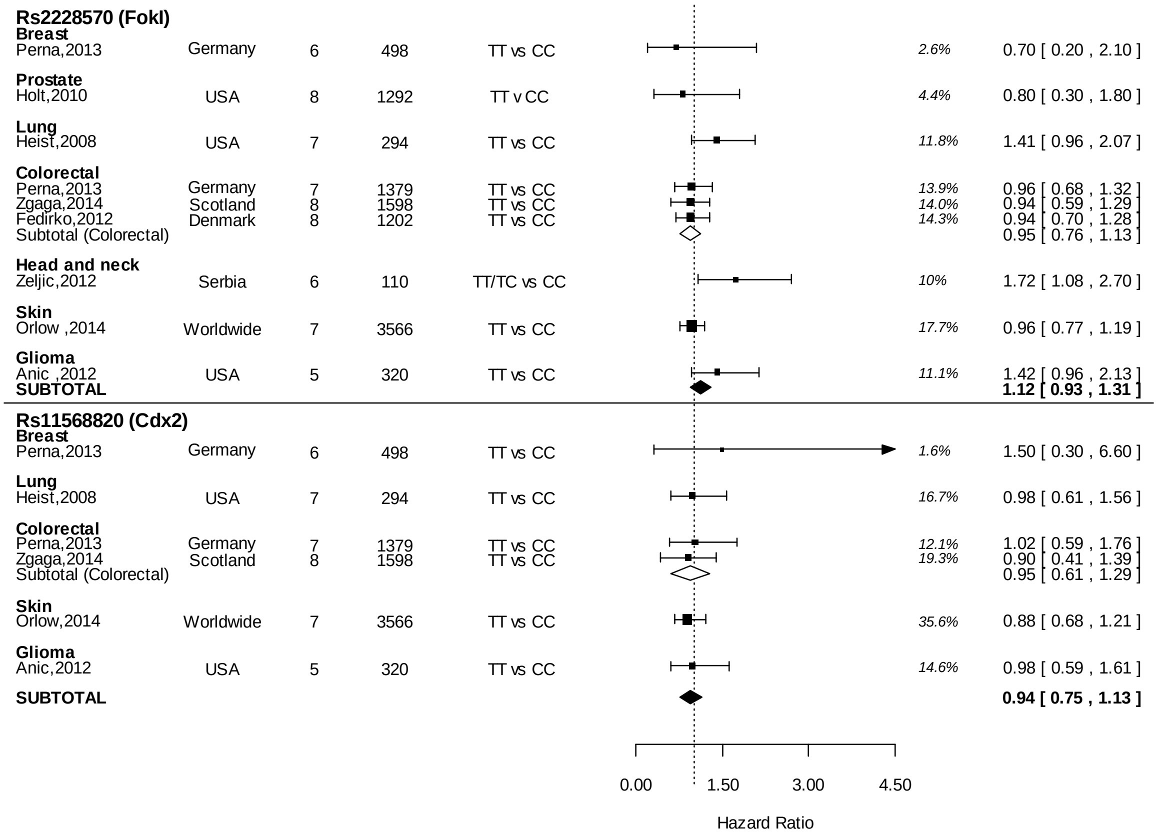
Rs2228570 (FokI)
Breast
Perna,2013 Germany 6 498 TT vs CC 2.6% 0.70 [0.20 , 2.10]
Prostate
Holt,2010 USA 8 1292 TT v CC 4.4% 0.80 [0.30 , 1.80]
Lung
Heist,2008 USA 7 294 TT vs CC 11.8% 1.41 [0.96 , 2.07]
Colorectal
Perna,2013 Germany 7 1379 TT vs CC 13.9% 0.96 [0.68 , 1.32]
Zgaga,2014 Scotland 8 1598 TT vs CC 14.0% 0.94 [0.59 , 1.29]
Fedirko,2012 Denmark 8 1202 TT vs CC 14.3% 0.94 [0.70 , 1.28]
Subtotal (Colorectal) 0.95 [0.76 , 1.13]
Head and neck
Zeljic,2012 Serbia 6 110 TT/TC vs CC 10% 1.72 [1.08 , 2.70]
Skin
Orlow ,2014 Worldwide 7 3566 TT vs CC 17.7% 0.96 [0.77 , 1.19]
Glioma
Anic ,2012 USA 5 320 TT vs CC 11.1% 1.42 [0.96 , 2.13]
SUBTOTAL 1.12 [0.93 , 1.31]
Rs11568820 (Cdx2)
Breast
Perna,2013 Germany 6 498 TT vs CC 1.6% 1.50 [0.30 , 6.60]
Lung
Heist,2008 USA 7 294 TT vs CC 16.7% 0.98 [0.61 , 1.56]
Colorectal
Perna,2013 Germany 7 1379 TT vs CC 12.1% 1.02 [0.59 , 1.76]
Zgaga,2014 Scotland 8 1598 TT vs CC 19.3% 0.90 [0.41 , 1.39]
Subtotal (Colorectal) 0.95 [0.61 , 1.29]
Skin
Orlow,2014 Worldwide 7 3566 TT vs CC 35.6% 0.88 [0.68 , 1.21]
Glioma
Anic,2012 USA 5 320 TT vs CC 14.6% 0.98 [0.59 , 1.61]
SUBTOTAL 0.94 [0.75 , 1.13]
0.00
1.50
3.00
4.50
Hazard Ratio

Adjusted Meta-analysis: Survival for Vitamin D receptor and pathway polymorphisms (continued)

Author and Year	Population	N.O. score	N	Exposure	Weight	HR [95% CI]
**Rs731236 (TaqI)**						
**Breast**						
Perna, 2013	Germany	6	498	GG vs AA	*10.7%*	3.00 [1.10 , 8.10]
**Prostate**						
Holt, 2010	USA	8	1292	GG vs AA	*11.1%*	0.50 [0.20 , 1.30]
**Colorectal**						
Perna, 2013	Germany	7	1379	GG vs AA	*21.4%*	0.79 [0.57 , 1.12]
**Head and neck**						
Zeljic, 2011	Serbia	6	110	AG/GG vs AA	*12.7%*	1.37 [0.90 , 2.10]
**Skin**						
Orlow , 2014	Worldwide	7	3566	GG vs AA	*23.6%*	0.81 [0.67 , 0.99]
**Glioma**						
Anic, 2012	USA	5	320	GG vs AA	*20.6%*	1.32 [0.88 , 1.96]
**Subtotal**						**1.18 [0.75 , 1.61]**
**Rs2296241 (CYP24A1(1))**						
**Head and neck**						
Zeljic, 2012	Serbia	6	110	AG/AA vs GG	*100%*	0.65 [0.38 , 1.11]
**Rs6013897 (CYP24A1(2))**						
**Glioma**						
Anic, 2012	USA	5	320	AA vs GG	*100%*	0.62 [0.40 , 0.96]
**Rs2282679 (GC)**						
**Glioma**						
Anic, 2012	USA	5	320	AA vs CC	*100%*	1.04 [0.69 , 1.54]
**Rs2107301**						
**Glioma**						
Anic , 2012	USA	5	320	CC vs TT	*22.1%*	1.02 [0.67 , 1.54]
**Skin**						
Orlow , 2014	Worldwide	7	3566	CC vs TT	*77.9%*	0.90 [0.74 , 1.12]
**Subtotal**						**0.93 [0.73 , 1.12]**
**Rs4516035 (EcoRV)**						
**Glioma**						
Anic, 2012	USA	5	320	TT vs CC	*100%*	1.25 [0.86 , 1.85]
**Rs2238135**						
**Glioma**						
Anic, 2012	USA	5	135	CC vs GG	*16.8%*	0.94 [0.58 , 1.49]
**Skin**						
Orlow , 2014	Worldwide	7	3566	CC vs GG	*83.2%*	0.96 [0.78 , 1.18]
**Subtotal**						**0.96 [0.77 , 1.14]**

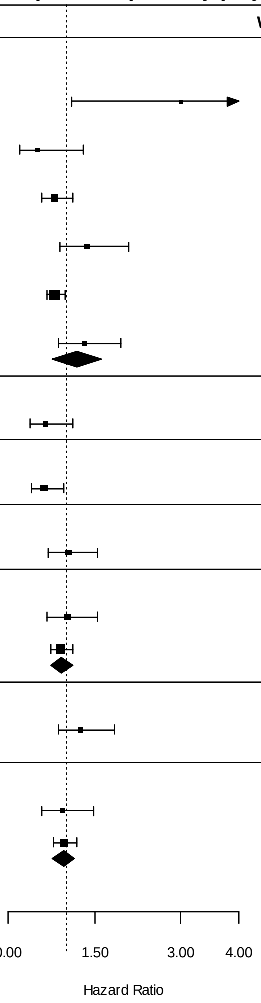

Supplement: Supplementary file 7 [file bjc201744x5.docx]
