# Supplementary material for: The impact of vitamin D pathway genetic variation and circulating 25-hydroxyvitamin D on cancer outcome: systematic review and meta-analysis
Source: Br J Cancer. 2017 Mar 16;116(8):1092–110. doi: 10.1038/bjc.2017.44 (PMC5396104; doi:10.1038/bjc.2017.44)

Adjusted Meta-analysis: Disease progression for Vitamin D receptor and pathway polymorphisms

Author and Year	Population	N.O. score	N	Exposure	Weight	HR [95% CI]
**Rs7975232 (ApaI)**						
**Prostate**						
Pao, 2013	Taiwan	8	601	AA vs CC	*100%*	1.31 [0.94 , 1.84]
**Rs1544410 (BsmI)**						
**Prostate**						
Pao, 2013	Taiwan	8	601	TT vs CC	*3.9%*	1.00 [0.14 , 7.21]
Williams, 2004	USA	7	728	TT vs CC	*33.3%*	0.60 [0.31 , 1.18]
Holt, 2010	USA	8	1294	TT vs CC	*62.8%*	1.10 [0.60 , 1.80]
**SUBTOTAL**						**0.93 [0.54 , 1.32]**
**Rs2228570 (FokI)**						
**Prostate**						
Pao, 2013	Taiwan	8	601	TT vs CC	*84.4%*	0.99 [0.74 , 1.33]
Holt, 2010	USA	8	1292	TT vs CC	*15.6%*	1.01 [0.60 , 2.00]
**SUBTOTAL**						**0.99 [0.72 , 1.26]**
**Rs731236 (TaqI)**						
**Prostate**					*100%*	
Holt, 2010	USA	8	1292	GG vs AA		0.80 [0.50 , 1.40]

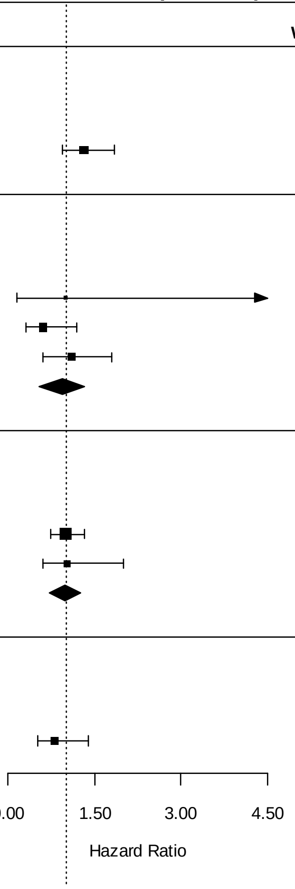

Supplement: Supplementary file 8 [file bjc201744x6.docx]
